# Macrophage‐Disguised Manganese Dioxide Nanoparticles for Neuroprotection by Reducing Oxidative Stress and Modulating Inflammatory Microenvironment in Acute Ischemic Stroke

**DOI:** 10.1002/advs.202101526

**Published:** 2021-08-26

**Authors:** Chao Li, Zhenhao Zhao, Yifan Luo, Tingting Ning, Peixin Liu, Qinjun Chen, Yongchao Chu, Qin Guo, Yiwen Zhang, Wenxi Zhou, Hongyi Chen, Zheng Zhou, Yu Wang, Boyu Su, Haoyu You, Tongyu Zhang, Xuwen Li, Haolin Song, Chufeng Li, Tao Sun, Chen Jiang

**Affiliations:** ^1^ Key Laboratory of Smart Drug Delivery Ministry of Education State Key Laboratory of Medical Neurobiology and MOE Frontiers Center for Brain Science Institutes of Brain Science Department of Pharmaceutics School of Pharmacy Fudan University Shanghai 201203 China

**Keywords:** biomimetic nanoparticles, inflammatory microenvironment, microglia polarization, neuroprotection, O_2_ generation, reperfusion injury, reactive oxygen species (ROS) consumption

## Abstract

Reperfusion injury is still a major challenge that impedes neuronal survival in ischemic stroke. However, the current clinical treatments are remained on single pathological process, which are due to lack of comprehensive neuroprotective effects. Herein, a macrophage‐disguised honeycomb manganese dioxide (MnO_2_) nanosphere loaded with fingolimod (FTY) is developed to salvage the ischemic penumbra. In particular, the biomimetic nanoparticles can accumulate actively in the damaged brain via macrophage‐membrane protein‐mediated recognition with cell adhesion molecules that are overexpressed on the damaged vascular endothelium. MnO_2_ nanosphere can consume excess hydrogen peroxide (H_2_O_2_) and convert it into desiderated oxygen (O_2_), and can be decomposed in acidic lysosome for cargo release, so as to reduce oxidative stress and promote the transition of M1 microglia to M2 type, eventually reversing the proinflammatory microenvironment and reinforcing the survival of damaged neuron. This biomimetic nanomedicine raises new strategy for multitargeted combined treatment of ischemic stroke.

## Graphical abstract

Macrophage‐disguised FTY‐loaded MnO_2_ nanoparticles (Ma@(MnO_2_+FTY)) are engineered to salvage the ischemic penumbra. Nanoparticles can accumulate actively in the ischemic region to protect neurons directly via consuming ROS and generating O_2_. In addition, the nanoparticles can also reverse the proinflammatory microenvironment by promoting the phenotypic transition of microglia through multiple signaling pathways, increasing the protection effects on damaged neurons.

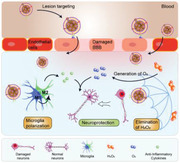

## 1 Introduction

Acute ischemic stroke, normally caused by the sudden obstruction of cerebral vessels by thrombus, is one of the leading causes of death and disability worldwide.^[^ ^1^ ^]^ Achieving reperfusion to salvage damaged neurons in ischemic penumbra is the main goal of most interventions.^[^ ^2^ ^]^ However, survivors who restored blood flow still face the risk of disability, mainly caused by secondary reperfusion injury following blood supply reconstruction.^[^ ^3^ ^]^ In addition, recanalization of occluded proximal arterial does not necessarily lead to the reperfusion of downstream microvessels where microthrombi are formed.^[^ ^4^ ^]^ Impaired microcirculation restricts the access of nearby neurons to necessary oxygen (O_2_) after the reperfusion, which further causes the neuronal death.^[^ ^5^ ^]^ Reducing reperfusion injury and minimizing the damage caused by microcirculation disorders remain major challenges in treating ischemic stroke.

Neurons are the most vulnerable cells compared with glia cells and vascular cells, and they rapidly lose normal function under ischemic condition.^[^ ^6^ ^]^ Ischemia and reperfusion can initiate cascade reactions, such as oxidative stress and inflammation response, which ultimately leads to the irreversible neuron injury.^[^ ^7^ ^]^ The overproduced reactive oxygen species (ROS) is a major cause of oxidative stress, and it leads to the neuronal necrosis or apoptosis through oxidizing bio‐macromolecules and activating apoptotic pathway.^[^ ^8^ ^]^ It has been proven that scavenging excessive ROS could effectively alleviate the progression of neuronal death and improve the prognosis.^[^ ^9^ ^]^ For instance, the free radical scavenger edaravone has shown effect clinically.^[^ ^10^ ^]^ However, for neurons in brain regions affected by microcirculation dysfunction, eliminating excess ROS alone is far from enough to maintain their homeostasis.^[^ ^11^ ^]^ O_2_ delivery systems based on nanotechnology, such as hemoglobin‐loaded liposomes, were utilized to salvage the ischemic brain in preclinical studies.^[^ ^12^ ^]^ Nevertheless, it is risky that delivering O_2_ imprecisely may cause unexpected ROS increase, as ROS is overproduced during ischemia and bursts due to the surge of O_2_ after reperfusion.^[^ ^13^ ^]^ The balance between ROS consumption and O_2_ delivery in rescuing neurons remains a high challenge. Manganese dioxide (MnO_2_) nanoparticles could consume excess hydrogen peroxide (H_2_O_2_) and convert it into O_2_ in situ, and this process is opposite to the abnormal ROS‐generation process.^[^ ^14^ ^]^ It is expected that the precise delivery of MnO_2_ nanoparticles to the ischemic brain could rescue the damaged neurons efficiently.

In addition, the damaged neurons would release the dangerous‐related molecular patterns which activate and recruit inherent microglia in the brain.^[^ ^15^ ^]^ M1 type (proinflammatory type) microglia would release the inflammatory mediators to construct the proinflammatory microenvironment, which would further destroy neurons.^[^ ^16^ ^]^ Numerous studies have shown that the functions of microglia are closely related with its phenotype, as promoting the polarization of microglia from M1 to M2 (anti‐inflammatory type) could attenuate the inflammation response and salvage the damaged neurons.^[^ ^17^ ^]^ Eliminating ROS has been proven to inhibit the secretion of proinflammatory cytokines from microglia by inhibiting the nuclear factor kappa light chain enhancer of activated B cells (NF‐*κ*B) pathway.^[^ ^18^ ^]^ In addition, some small molecule drugs, such as fingolimod (FTY) and curcumin, could also polarize microglia toward M2‐type in ischemic brain.^[^ ^19^ ^]^

The blood–brain barrier (BBB) restricts the entry of most therapeutic species into the brain, which hinders the treatment of brain diseases while maintaining brain homeostasis.^[^ ^20^ ^]^ The integrity of BBB is partly destroyed during the ischemia and reperfusion, which provides an opportunity for the therapeutic agents to reach the lesions.^[^ ^21^ ^]^ Nevertheless, the effect of nanomedicines based on the temporary opening of the BBB still needs further improvement, since the degree and timing of the opening of BBB vary greatly among individuals with different severity of ischemia.^[^ ^21a^ ^]^ During the ischemia and reperfusion, intercellular adhesion molecule‐1 (ICAM‐1) and P‐selectin are overexpressed on the lumen side of stressed vascular endothelial cells.^[^ ^22^ ^]^ These molecules can interact with corresponding ligands on leukocytes (CD44 and CD11b) recruited from peripheral blood and promote their infiltration into the lesions, and the recruitment is not affected by the openness of BBB.^[^ ^23^ ^]^ Recently, biomimetic nanomedicine coating with cell membrane have been extensively studied, the natural properties inherited from cell help nanoparticles reduce their immunogenicity and prolong the half‐life.^[^ ^24^ ^]^ Moreover, coating cell membrane origin from leukocytes has been shown to endow nanoparticles with accurate targeting capability to inflammatory lesions.^[^ ^25^ ^]^ Macrophages would be recruited and infiltrate into the ischemic brain within hours,^[^ ^26^ ^]^ and therefore coating with macrophage‐membrane is a promising strategy for the accurate delivery of nanoparticles to ischemic lesions.

**Scheme 1:**
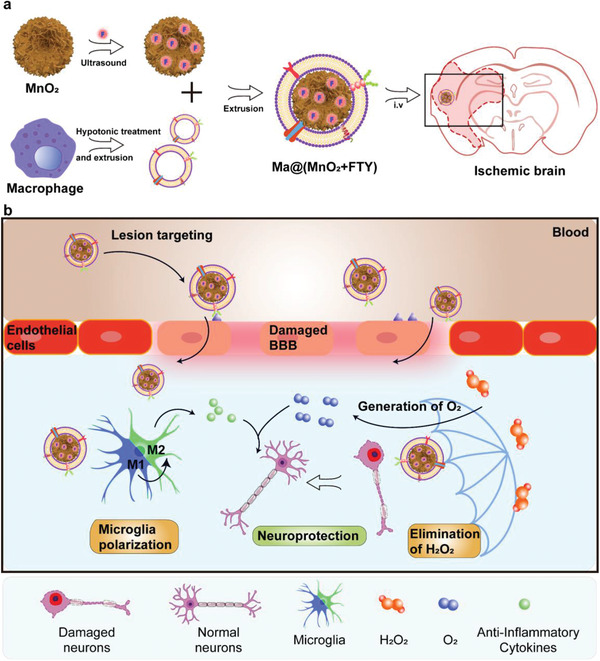
Illustration of Ma@(MnO_2_+FTY) nanoparticles formation and salvation of damaged neurons in ischemic brain. a) Scheme of the preparation process of Ma@(MnO_2_+FTY) nanoparticles. b) Illustration of Ma@(MnO_2_+FTY) therapy for the rescue of ischemic penumbra. Coating with macrophage cell membrane increased the accumulation of Ma@(MnO_2_+FTY) nanoparticles in the damaged brain. Ma@(MnO_2_+FTY) could protect damaged neurons by consuming ROS, generating O_2_ and regulating proinflammatory microenvironment through promoting the phenotypic transition of microglia.

Herein, macrophage‐disguised FTY‐loaded MnO_2_ nanoparticles (Ma@(MnO_2_+FTY)), with the ability to consume ROS, generate O_2_, and reverse the proinflammatory microenvironment, were engineered to salvage the ischemic penumbra (**Scheme** **1**). Coating with macrophage membrane endows the nanoparticles with inflammation‐oriented chemotactic abilities. MnO_2_ nanoparticles with high surface area could efficiently consume excessive ROS and generate O_2_ to rescue dying neurons. Consuming ROS can also inhibit the NF‐*κ*B signaling pathway in microglia, thereby to attenuate the proinflammatory response. FTY, encapsulated in MnO_2_ nanoparticles through the electrostatic interaction, promoted the phenotypic transition of microglia to reverse the proinflammatory microenvironment through activating the signal transducer and activator of transcription 3 (STAT3) pathway. The results indicated that the combination of reducing oxidative stress and regulating proinflammatory microenvironment could achieve synergistic neuroprotection effect in treating ischemic stroke.

## 2 Results and Discussion

### 2.1 Preparation and Characterization of Ma@(MnO_2_+FTY)

**Figure 1:**
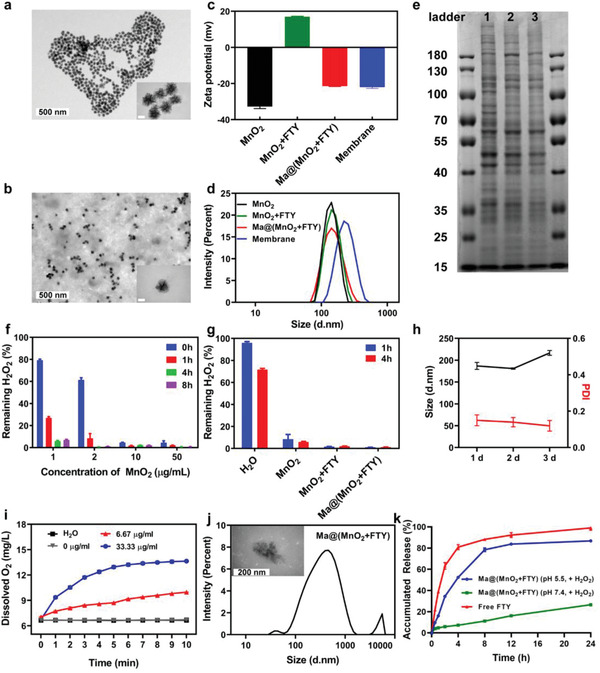
Characterizations of macrophage‐disguised MnO_2_ nanospheres. a) Representative TEM images of the honeycomb MnO_2_ nanospheres (inner scale bar, 50 nm). b) Representative TEM images of Ma@(MnO_2_+FTY) nanospheres (inner scale bar, 50 nm). c) Zeta‐potential and d) size distribution of as‐prepared MnO_2_, MnO_2_+FTY, and Ma@(MnO_2_+FTY) nanoparticles and macrophage cell membrane vesicles. e) Proteins in macrophage membrane, macrophage membrane vesicles and Ma@(MnO_2_+FTY), analyzed with SDS‐PAGE. f) H_2_O_2_ scavenging behavior of MnO_2_ nanospheres with multiple concentration over 8 hours. Data are presented as means ± SD, *n* = 3. g) H_2_O_2_ scavenging behavior of different formulations. Results are reported as means ± SD, *n* = 3. h) The size and PDI of Ma@(MnO_2_+FTY) in 10% FBS over 3 days. Results are presented as means ± SD, *n* = 3. i) The changes of O_2_ concentration in 100 × 10^−6^ m H_2_O_2_ after MnO_2_ with different concentration were added, the changes of O_2_ concentration in H_2_O were used as a control. Data are reported as means ± SD, *n* = 3. J) The size change and representative TEM images of Ma@(MnO_2_+FTY) after incubation in PBS 6.0 with 100 × 10^−6^ m H_2_O_2_ for 30 min at 37 °C (inner scale bar, 200 nm). k) The in vitro release profile of FTY from Ma@(MnO_2_+FTY) in the condition which mimics M1 microglia. The release profile of free FTY in CH_3_COONa buffer was used as a control. Data are presented as means ± SD, *n* = 3.

Honeycomb MnO_2_ nanospheres were prepared via a modified soft chemistry method.^[^ ^27^ ^]^ In brief, potassium permanganate (KMnO_4_) was reduced by oleic acid (OA) in distilled water at room temperature. OA played the roles as synthesis template and meanwhile reductant in this process, and MnO_2_ core grew on the surface of oil/water emulsion formed by OA. The UV–vis absorption spectra showed that the main peaks of KMnO_4_ (310, 524, and 544 nm) disappeared upon the reaction completion, and a new broad peak appeared at about 368 nm (Figure S1, Supporting Information), which indicated the formation of new species. The crystallographic structure and element composition of the prepared samples were analyzed by powder X‐ray diffraction (PXRD) and energy‐dispersive spectroscopy (EDS), respectively. As shown in the PXRD pattern (Figure S2, Supporting Information), main peaks recorded at 2*θ* = 12.15°, 23.75°, 36.54°, and 65.55° well corresponded to the (001), (002), (100), and (110) lattice planes of turbostatic MnO_2_.^[^ ^28^ ^]^ The results of EDS measurement validated the atomic ratio of Mn/O was about 1:2 (Figure S3, Supporting Information). Scanning electron microscopy (SEM) and transmission electron microscopy (TEM) images showed that as‐prepared MnO_2_ possessed a honeycomb spherical structure (**Figure** **1**a; Figure S4, Supporting Information). The size of such nanoparticles self‐assembled by several platelets was calculated to be around 120 nm in a magnified image (inset in Figure 1a). We next determined the specific surface area and pore size distribution of honeycomb MnO_2_ nanospheres via a classic N_2_ adsorption–desorption evaluation. The results showed that the BET surface area of MnO_2_ was about 6.75 m^2^ g^−1^, and the pore size mainly distributed at 4.3 nm (Figures S5 and S6, Supporting Information). The large surface area and abundant pores provides more reactive sites to the contact and consumption of H_2_O_2_, and the negatively charged surface is beneficial to loading positively charged drugs via electrostatic adsorption.^[^ ^29^ ^]^

We then explored the drug loading capacity of MnO_2_. FTY was added into the MnO_2_ colloid and ultrasonicated for 1 h to achieve successful loading, and excess drug was removed by centrifugation. The loading efficiency determined by high‐performance liquid chromatography (HPLC) was about 33.3%. To improve the stability and targeting ability of above nanoparticles, rat peritoneal macrophage cell membrane was adopted and extruded to obtain corresponding vesicles. FTY‐loaded MnO_2_ (MnO_2_+FTY) was coextruded with macrophage‐membrane vesicles to construct the macrophage‐membrane‐coated FTY‐loaded MnO_2_ (Ma@(MnO_2_+FTY)) nanoparticles. The ratio of cell membrane (quantified by protein content) to MnO_2_+FTY was optimized by monitoring the change of zeta‐potential values. A cell membrane protein to MnO_2_+FTY weight ratio of 2:1 produced stable Ma@(MnO_2_+FTY) with a zeta‐potential near to that of membrane vesicle (Figure S7, Supporting Information). The properties of the prepared nanoparticles were then investigated with dynamic light scattering (DLS) and nanoparticle tracking analysis (NTA). The zeta‐potential of MnO_2_, MnO_2_+FTY, Ma@(MnO_2_+FTY) and membrane vesicles were −32.77 ± 1.10, 16.97 ± 0.31, −21.43 ± 0.23, and −22.00 ± 0.69 mV, respectively (Figure 1c). The hydrodynamic diameter of MnO_2_, MnO_2_+FTY, Ma@(MnO_2_+FTY) and the membrane vesicles were about 122, 123, 144, and 220 nm, respectively (Figure 1d; Figure S8, Supporting Information). The particle size of Ma@(MnO_2_+FTY) was about 20 nm that is higher than that of MnO_2_, and the zeta potential of Ma@(MnO_2_+FTY) was almost close to that of the membrane vesicles, revealing the successful wrapping of macrophage‐membrane onto the surface of MnO_2_ nanospheres. Furthermore, the results of TEM also indicated the successful coating, since there was a distinct shell on the surface of MnO_2_ core in the magnified image (inset of Figure 1b).

Since the cell membrane surface proteins play an important role in the process of lesions targeting, we further investigated the changes of surface cell membrane proteins on nanoparticles after coextrusion. Membrane proteins analyzed by SDS‐PAGE indicated that Ma@(MnO_2_+FTY) retained most cell membrane surficial proteins (Figure 1e), including the key proteins (CD44 and CD11b) involved in targeting effect (Figure S9, Supporting Information). In order to further confirm that the correct orientation of the cell membrane was retained after the coextrusion, we performed immunostaining of the extracellular domain of the cell membrane protein CD11b. There was significant attachment of the gold particles to Ma@(MnO_2_+FTY) nanoparticles, compared to the negative control (Figure S10, Supporting Information). These results confirmed the presence of right‐side‐out of membrane on the Ma@(MnO_2_+FTY) nanoparticles. We next studied the stability of nanoparticles under simulated physiological condition. Naked nanoparticles were difficult to maintain stable in saline or phosphate buffer, while the tactic of coating with cell membrane improved the dispersion and stability of the nanoparticles in solutions (Figure S11a, Supporting Information). Moreover, the hydrodynamic diameter and polydispersity index (PDI) of Ma@MnO_2_ and Ma@(MnO_2_+FTY) in PBS or 10% FBS demonstrated the stability of the prepared nanomedicine under physiological conditions (Figure 1h; Figure S11b,c, Supporting Information).

### 2.2 ROS Scavenging and O_2_‐Producing Properties of Ma@(MnO_2_+FTY)

The overproduced ROS, including superoxide anion, H_2_O_2_, and hydroxyl radical, would cause abnormally elevated level of oxidative stress and undue neuronal injury after the ischemia and reperfusion.^[^ ^30^ ^]^ At the same time, it is difficult for the neurons affected by microcirculation disorders to obtain enough O_2_ after the reperfusion, which further leads to the neuronal death.^[^ ^5^ ^]^ As we all know, MnO_2_ could catalyze H_2_O_2_ into O_2_, and this process is opposite to the abnormal ROS‐production process. We then investigated the ability of MnO_2_ to eliminate H_2_O_2_ and generate O_2_. The ROS eliminating ability of MnO_2_ was detected through quantifying the residual H_2_O_2_ after being incubated with different concentrations of MnO_2_ for specific times. The results showed that MnO_2_ could efficiently scavenge H_2_O_2_ and the elimination process is concentration‐ and time‐dependent (Figure 1f). It is worth mentioning that only 1 µg mL^−1^ MnO_2_ eliminated nearly 80% of H_2_O_2_ within 1 h. Meanwhile, the loading of FTY and coating with cell membrane did not interfere with the H_2_O_2_ scavenging ability of MnO_2_ nanoparticles. Interestingly, the scavenging efficacy of Ma@(MnO_2_+FTY) increased compared with that of naked MnO_2_, which might be attributed to the reaction between cell membrane components and H_2_O_2_ (Figure 1g). In addition, we found the significant production of O_2_ after adding different concentration of MnO_2_ into H_2_O_2_ solution, accompanied with the degradation of H_2_O_2_ (Figure 1i; Figure S12, Supporting Information). The above results indicated that MnO_2_ is expected to play potential therapeutic effect by scavenging ROS and generating O_2_ in the ischemic brain after the reperfusion.

In addition, MnO_2_ could also react with H_2_O_2_ and H^+^ to be decomposed into divalent manganese ions (Mn^2+^) and produce O_2_.^[^ ^31^ ^]^ We speculate that the nanoparticles would disintegrate and release the loaded drug after being internalized into cells and translocated into the acidic lysosomes. Buffer solution that mimics the lysosomal environment of the stressed cell under ROS was applied to verify this hypothesis. We found the particle size of Ma@(MnO_2_+FTY) changed significantly after being incubated in PBS 6.0 containing 100 × 10^−6^ m H_2_O_2_ at 37 °C for 30 min (Figure 1j). The TEM images also confirmed the disintegration of Ma@(MnO_2_+FTY) nanoparticles, since the shape of the nanoparticles changed from honeycomb to irregular lamella (Figure 1j; Figure S13, Supporting Information). The disintegration of Ma@(MnO_2_+FTY) nanoparticles led to the responsive release of FTY (Figure 1k), which is conducive to the intracellular regulation of the STAT3 pathway. Moreover, the generated paramagnetic Mn^2+^ could act as the contrast agent for enhanced T1 magnetic resonance imaging (MRI).^[^ ^32^ ^]^ MRI images showed the obvious concentration‐dependent brightening behavior of Ma@(MnO_2_+FTY) in PBS 6.0 containing 100 × 10^−6^ m H_2_O_2_, however, the signals in PBS 7.4 were much weaker (Figure S14a, Supporting Information). Importantly, the T1 relaxation rate (1/T1) raised linearly with the concentration of Mn^2+^, and the transverse relativity r1 value significantly increased from 3.745 mm ^−1^ s^−1^ in PBS 7.4 to 54.30 mm ^−1^ s^−1^ after the incubation in PBS 6.0 for 12 h, which was attributed to the decomposition from MnO_2_ into Mn^2+^ (Figure S14b, Supporting Information). These results indicated that MnO_2_ nanoparticles could be employed to image the ischemic cerebral lesions after the reperfusion.

### 2.3 The Neuroprotective Effects of Ma@(MnO_2_+FTY)

**Figure 2:**
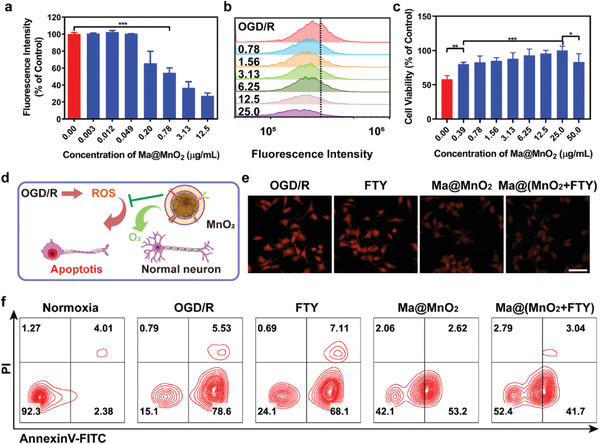
Neuroprotection effect of macrophage membrane‐coated MnO_2_ nanospheres. a) Relative quantification of the green fluorescence intensity in SH‐SY5Y cells with OGD/R treatment. Results are reported as means ± SD, *n* = 3, ^***^ *P* < 0.001. b) The generation of O_2_ in SH‐SY5Y cells treated with OGD/R, cells were incubated with Ma@MnO_2_ of different concentrations (unit: µg mL^−1^) and analyzed with flow cytometry. [(Ru(dpp)_3_)]Cl_2_, a fluorescence probe which could be quenched by O_2_, was applied to detect the production of O_2_. c) The cell viability of SH‐SY5Y cells after OGD/R or incubated with Ma@MnO_2_ of different concentration for 24 hours. Data are presented as means ± SD, *n* = 5, ^**^ *P* < 0.01. d) Scheme of the mechanism of neuroprotection by Ma@MnO_2_ nanospheres on damaged neurons suffered OGD/R. e) Representative images of generated O_2_ in SH‐SY5Y cells which were treated with OGD/R and incubated with different formulations for 12 h (scale bar, 50 µm). f) Representative images of cell apoptosis analyzed with flow cytometry, where SH‐SY5Y cells were treated with OGD/R, and incubated with different formulations.

Since ROS is one of the main sources of neuronal damage, we then investigated the neuroprotective effects and proposed potential mechanism of neuroprotection by Ma@(MnO_2_+FTY) nanospheres in vitro (**Figure** **2**d). SH‐SY5Y cells treated with oxygen‐glucose deprivation/reoxygenation (OGD/R) were employed to mimic neurons under ischemia and reperfusion. The results of the cell viability experiment confirmed that the Ma@MnO_2_ nanoparticles elicited no obvious cytotoxicity on SH‐SY5Y cells even at 12.5 µg mL^−1^ (Figure S15, Supporting Information). The level of ROS detected by 2′,7′‐dichlorodihydrofluorescein diacetate (H_2_DCFDA) increased drastically in neurons after OGD/R, and the treatment with Ma@MnO_2_ reduced the green fluorescence intensity in a concentration‐dependent manner (Figure 2a; Figure S16, Supporting Information). We then investigate the generation of O_2_ with a O_2_ sensing probe ([(Ru(dpp)_3_)]Cl_2_) in SH‐SY5Y cells. The level of O_2_ in stressed neurons improved significantly after being incubated with Ma@MnO_2_ of different concentrations (Figure 2b; Figure S17, Supporting Information). At the same time, treatment with FTY did not promote the production of O_2_, and the loading of FTY did not affect the O_2_ production ability of Ma@MnO_2_ nanoparticles (Figure 2e). The reduction in the level of oxidative stress and the generation of O_2_ promoted the survival of neurons, as the viability of SH‐SY5Y cells increased remarkably (Figure 2c). Interestingly, neurons suffered OGD/R showed stronger tolerance than the normal neurons when being treated with Ma@MnO_2_ at 25 µg mL^−1^, and this phenomenon might be attributed to the rapid conversion of ROS into less toxic substances by the high concentration of Ma@MnO_2_. Moreover, the results of the flow cytometry analysis showed that neurons underwent severe apoptosis after OGD/R, where both Ma@MnO_2_ and Ma@(MnO_2_+FTY) significantly rescued neurons from apoptosis caused by the oxidative stress (Figure 2f). The combination of these results revealed that Ma@MnO_2_ and Ma@(MnO_2_+FTY) could directly protect the neurons from damage by converting ROS into O_2_ and reducing the level of oxidative stress.

### 2.4 Ma@(MnO_2_+FTY) Promoted Microglia Polarization from M1 to M2 In Vitro

As the inherent immune cells in the brain, microglia are extremely sensitive to changes in the microenvironment, and will be quickly activated and recruited to the lesions after the ischemia.^[^ ^15^ ^]^ The M1 type predominates in the acute phase of ischemia, and it promotes the formation of proinflammatory microenvironment by increasing the level of oxidative stress and secreting inflammatory factors, thereby aggravating the damage to neurons.^[^ ^16^ ^]^ Mediating the polarization of microglia to M2 could increase the secretion of anti‐inflammatory cytokines, weaken the inflammatory response, and exert a protective effect on the damaged neurons.^[^ ^17^ ^]^ It is reported that the ceria nanoparticles could promote the polarization of microglia from M1 to M2 by scavenging ROS, and this process involved the ROS‐triggered NF‐*κ*B signaling pathway.^[^ ^18^ ^]^ In addition, FTY was also reported able to regulate the phenotypic transition of M1 microglia to M2 type via promoting the upregulation of phosphorylated‐STAT3 (p‐STAT3).^[^ ^19a^ ^]^ Therefore, we then conducted a series of in vitro experiments to explore the ability of Ma@(MnO_2_+FTY) nanoparticles to promote the phenotype transformation of microglia from M1 to M2 to exert the anti‐inflammatory effects.

**Figure 3:**
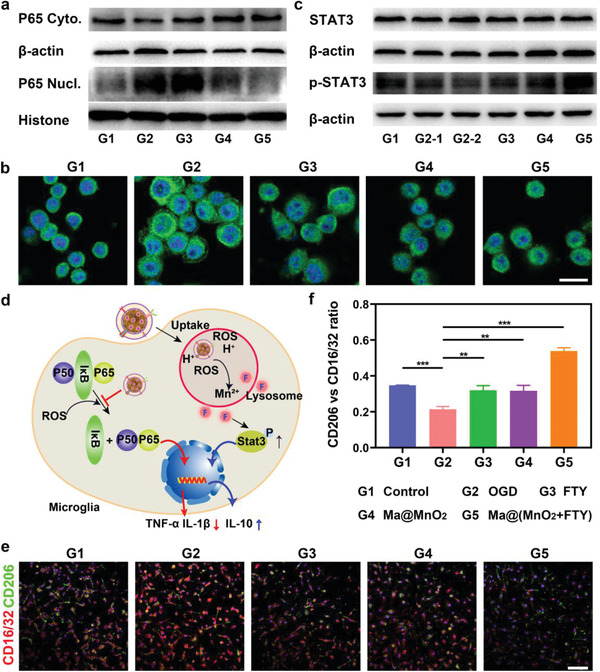
Modulation of microglia phenotype and the potential mechanism of action by Ma@(MnO_2_+FTY) nanoparticles. a) Expression of P65 in the cytoplasm and nucleus of microglia with different treatment, characterized by western blotting. b) Representative images of p‐P65 in microglia with different treatment which attenuated the expression of p‐P65 and reduced the transfer to the nucleus (scale bar, 25 µm). c) Expression of STAT3‐associated protein in microglia with different treatment. d) Scheme of the mechanism for microglia polarization by Ma@(MnO_2_+FTY) nanoparticles. e) Representative images of microglia immunostained with CD16/32 and CD206 (scale bar, 100 µm). f) Quantification of the relative fluorescence intensity of CD206 versus CD16/32. Data are presented as means ± SD, *n* = 3, ^**^ *P* < 0.01.

Firstly, the results of the cell viability experiments confirmed that the Ma@MnO_2_ nanoparticles elicited no obvious cytotoxicity on BV2 cells at 12.5 µg mL^−1^ (Figure S18a, Supporting Information). It is worth mentioning that the increased cytotoxicity of Ma@(MnO_2_+FTY) was attributed to the loaded FTY (Figure S18b,c, Supporting Information), the concentration of nanoparticles used in subsequent experiments was in a safe range. The uptake inhibition experiment with flow cytometry revealed the internalization of Ma@(MnO_2_+FTY) in BV2 cells mainly through the energy‐dependent caveolin‐mediated endocytic pathway (Figure S19, Supporting Information). The cellular uptake results characterized by confocal imaging and flow cytometry showed that the uptake of Ma@MnO_2_ and Ma@(MnO_2_+FTY) by BV2 cells was time‐dependent (Figure S20b, Supporting Information), and the nanoparticles were found colocalized with lysosomes after being internalized into cells, which was conducive to the decomposition of nanoparticles and the release of loaded FTY (Figure S20a, Supporting Information). In addition, treatment with Ma@(MnO_2_+FTY) markedly reduced the level of ROS in BV2 cells subjected to OGD/R (Figure S21, Supporting Information), which was expected promote the phenotype transformation of microglia from M1 to M2 through scavenging ROS. In order to better investigate the microglial phenotypic transition effect of nanoparticles, we isolated and purified the primary microglia for further studies (Figure S22, Supporting Information). Microglia undergoing OGD/R were treated with different drugs, and the status of primary microglia were monitored by investigating the expression of M1 marker CD16/32 and M2 marker CD206. The immunofluorescence results showed that OGD/R led to significantly elevated expression of CD16/32, and both free FTY and Ma@MnO_2_ nanoparticles reduced the level of CD16/32 and increased the expression of CD206 (**Figure** **3**e). The effect of Ma@(MnO_2_+FTY) further increased, as the semi‐quantitative results of the immunofluorescence showed a significant enhance in the ratio of M2/M1 (Figure 3f). We then explored the potential mechanisms of microglial transformation in vitro by the immunofluorescence and western blot. It is reported that ROS could lead the activation of NF‐*κ*B signaling pathway, and the key protein (P65 and P50) would form heterodimer and translocate into the nucleus to activate a series of inflammatory response.^[^ ^18^ ^]^ We found that P65 translocated from the cytoplasm to the nucleus in microglia after OGD/R, and treated with both Ma@MnO_2_ and Ma@(MnO_2_+FTY) nanoparticles reversed this process (Figure 3a). At the same time, fluorescence imaging results showed that the level of phosphorylated‐P65 (p‐P65) increased, and p‐P65 mainly distributed in the nucleus and its surroundings in microglia (Figure 3b). Both Ma@MnO_2_ and Ma@(MnO_2_+FTY) nanoparticles could decrease the expression of p‐P65 and reduce the distribution in nucleus. In addition, the p‐STAT3 level decreased in microglia after OGD/R, while both the free FTY and nanoparticles increased its expression (Figure 3c). The combination of these results revealed that Ma@(MnO_2_+FTY) nanoparticle could promote the transformation of microglia from M1 to M2 through multiple signaling pathways, where the mechanism of microglia polarization by Ma@(MnO_2_+FTY) nanoparticles was shown in Figure 3d.

### 2.5 Targeting Capability of Ma@(MnO_2_+FTY) to the Ischemic Brain In Vivo

**Figure 4:**
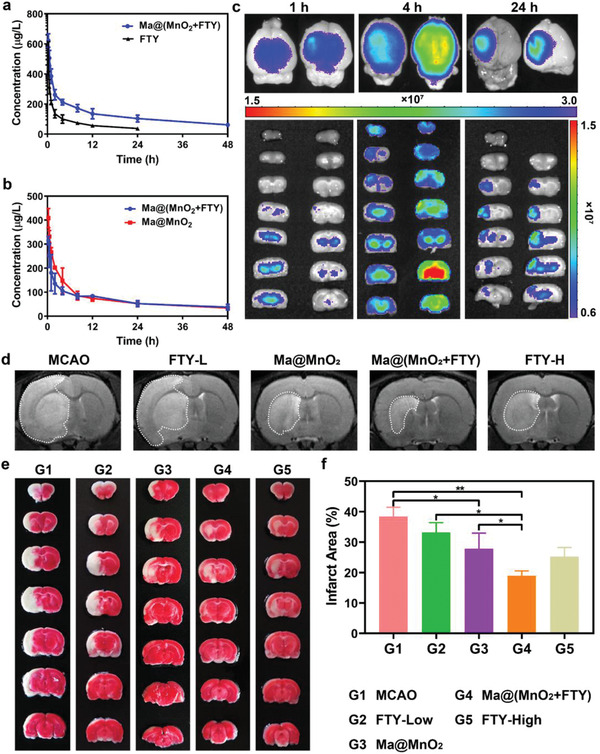
The pharmacokinetic behavior, targeting capability, and therapeutic effect of as‐prepared nanomedicine in vivo. a) The pharmacokinetic profiles of FTY after intravenous injection of free FTY and Ma@(MnO_2_+FTY) nanoparticles with an equal FTY dose of 1.5 mg kg^−1^. Data are presented as means ± SD, *n* = 3. b) The pharmacokinetic profiles of MnO_2_ after intravenous injection of Ma@MnO_2_ and Ma@(MnO_2_+FTY) nanoparticles at an equal MnO_2_ dose of 3 mg kg^−1^. Results are reported as means ± SD, *n* = 3. c) The targeting results of Ma@(MnO_2_+FTY) nanoparticles to the ischemic brain, where fluorescence intensity in ischemic brain was observed at different times after injection of labeled nanoparticles. d) The infarct area of tMCAO/R rats treated with different drugs, monitored by MRI at 24 h post reperfusion. e) The rescue ability of nanoparticles on ischemic penumbra, brain sections were stained with TTC. f) The quantified results of TTC staining. Data are presented as means ± SD, *n* = 3, ^*^ *P* < 0.05, ^**^ *P* < 0.01.

Coating macrophage cell membrane onto the surface of nanocarriers has been reported to confer them with natural properties from the original cell membrane, which not only reduced the immunogenicity and prolonged their half‐life, but also conferred them accurate targeting capability.^[^ ^24^, ^25^ ^]^ The half‐life of free FTY measured by LC/MS‐MS was about 14.597 ± 1.623 h, encapsulating in Ma@MnO_2_ could significantly prolong the half‐life of FTY to 30.208 ± 2.804 h (**Figure** **4**a). Meanwhile, loading with drugs did not significantly change the half‐life of Ma@MnO_2_ in vivo (Figure 4b). Prolonged circulation time provides more opportunities for nanoparticles to enter the ischemic lesions via the interaction between membrane proteins and cell adhesion molecule that is highly expressed on the damaged vascular endothelium. We further assessed the targeting capability of Ma@(MnO_2_+FTY) nanoparticles in a rat model of transient middle cerebral artery occlusion/reperfusion (tMCAO/R). Fluorescence of the probe in ischemic brain was observed at different times after the injection with nanoparticles. Ma@(MnO_2_+FTY) accumulated prominently in the ischemic brain compared with free probe at 4 h after the injection, and there was still significant retention at 24 h post injection (Figure 4c). We further performed tissue distribution experiment to confirm the targeting ability of Ma@(MnO_2_+FTY) nanoparticles to cerebral ischemic lesions. In order to maintain the consistency of particle size and zeta‐potential, erythrocyte membrane‐coated FTY‐loaded MnO_2_ nanoparticles (RM@(MnO_2_+FTY)) were applied as a control. As shown in Figure S23 (Supporting Information), compared with the RM@(MnO_2_+FTY) group, the accumulation of the Ma@(MnO_2_+FTY) nanoparticles in the ischemic lesion was significantly increased. These results confirmed that the Ma@(MnO_2_+FTY) nanoparticles possess well ischemic region targeting ability in addition to the prolonged circulation lifetime. In addition, the T1‐weighted MRI images enhanced by Mn^2+^ also confirmed the targeting capability of the nanoparticles, since the signal brightness of damaged striatum increased after the intravenous injection with Ma@(MnO_2_+FTY) for 4 h (Figure S24, Supporting Information).

### 2.6 Ma@(MnO_2_+FTY) Relieved the Brain Damage and Reversed the Proinflammatory Microenvironment

Therefore, we then evaluated the ischemic penumbra protection efficacy of nanoparticles on the rat models of tMCAO/R. The rats were randomly divided into five groups after a 2 h reperfusion and injected intravenously with saline (MCAO), 1.5 mg kg^−1^ FTY (FTY‐Low), 3 mg kg^−1^ FTY (FTY‐High), and Ma@MnO_2_ and Ma@(MnO_2_+FTY) (with an equal dose of 3 mg kg^−1^ MnO_2_), and the dose of FTY in Ma@(MnO_2_+FTY) group was 1.5 mg kg^−1^. The infarct area was monitored by MRI at 24 h post reperfusion. The rats were then sacrificed, the brain sections were stained with 2,3,5‐triphenyltetrazolium chloride (TTC) and quantified to investigate the rescue ability of nanoparticles on ischemic penumbra. Both the MRI and TTC staining results validated the significant protection efficacy of Ma@(MnO_2_+FTY) on the ischemic brain (Figure 4d–f, Supporting Information).

**Figure 5:**
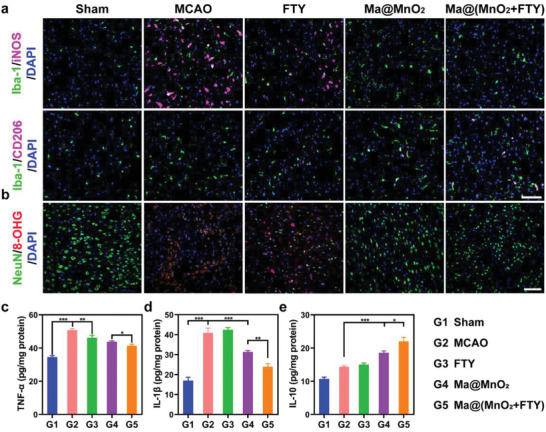
The modulation of proinflammatory microenvironment by Ma@(MnO_2_+FTY) nanoparticles in the ischemic brain. a) Treatment with Ma@(MnO_2_+FTY) could significantly reduce the population of M1 microglia (iNOS^+^Iba‐1^+^ cells) and increase the population of M2 microglia (CD206^+^Iba‐1^+^ cells), indicating the phenotypic transition of microglia from M1 to M2 (scale bar, 100 µm). b) The level of oxidative stress (8‐OHG) of damaged neurons decreased markedly and the number of neurons (NeuN) increased significantly (scale bar, 25 µm). c–e) Treatment with Ma@(MnO_2_+FTY) nanoparticles reversed the proinflammatory microenvironment. The expression of proinflammatory cytokines including c) TNF‐*α* and d) IL‐1*β* decreased, and the level of anti‐inflammatory cytokines e) IL‐10 increased. Data are presented as means ± SD, *n* = 3, ^*^ *P* < 0.05, ^**^ *P* < 0.01, ^***^ *P* < 0.001.

We then explored the capability of Ma@(MnO_2_+FTY) to reverse the proinflammatory microenvironment to salvage damaged neurons. The immunostaining of ischemic brain tissue showed an obvious increase of iNOS^+^Iba‐1^+^ cells in tMCAO/R rats compared with the sham group. Treatment with Ma@(MnO_2_+FTY) could apparently reduce the population of M1 microglia and increase the population of M2 microglia, and this phenomenon was consistent with what was observed in vitro (**Figure** **5**a). In addition, flow cytometry study also showed that Ma@(MnO_2_+FTY) could promote the polarization of microglia from M1 to M2 in the damaged brain of tMCAO/R rats (Figure S25, Supporting Information). Moreover, treating with Ma@(MnO_2_+FTY) could reduce the expression of p‐P65 in activated microglia in vivo (Figures S26 and S27, Supporting Information).The phenotypic transition of microglia from M1 to M2 could decrease the expression of proinflammatory cytokines including TNF‐*α* and IL‐1*β* (Figure 5c,d), increase the secretion of anti‐inflammatory cytokines IL‐10 (Figure 5e), which promoted the formation of anti‐inflammatory microenvironment. As a result, the level of oxidative stress of neurons decreased markedly and the number of neurons increased significantly, indicating the protective efficacy of anti‐inflammatory microenvironment on damaged neurons (Figure 5b). These results validated that treatment with Ma@(MnO_2_+FTY) could achieve synergistic neuroprotective effect in treating ischemic stroke through the combination of reducing oxidative stress and regulating inflammatory microenvironment. The neurological assessment was then performed with animal behavior test to confirm the improvement of motor function of tMCAO/R rats. The results showed that treatment with Ma@(MnO_2_+FTY) significantly reduced the behavioral defects in model rats (Figure S28, Supporting Information).

### 2.7 The Safety of Ma@(MnO_2_+FTY) Nanoparticles

We finally investigated the safety of the nanoparticles in vivo and in vitro. The results of hemolysis test showed that coating with cell membranes could significantly attenuate the hemolysis caused by positively charged FTY‐loaded MnO_2_, which validated the hemocompatibility of the nanoparticles (Figure S29, Supporting Information). It has been reported that the use of FTY may cause a temporary increase in alanine aminotransferase (ALT) and aspartate aminotransferase (AST),^[^ ^33^ ^]^ which is consistent with our findings. Ma@MnO_2_ did not cause such side effects, at the same time, encapsulating FTY in honeycomb MnO_2_ prevented the abnormal elevation of ALT and AST (Figure S30, Supporting Information). In addition, the hematoxylin–eosin (H&E) staining results of tissue sections showed no significant abnormalities, indicating that the intravenous administration of nanomedicine did not cause any organ toxicity (Figures S31 and S32, Supporting Information). The combination of these results revealed the favorable safety of as‐prepared nanomedicine.

## 3 Conclusion

In conclusion, we developed a macrophage‐disguised FTY‐loaded manganese dioxide nanoparticles to protect neurons through reducing oxidative stress and regulating proinflammatory microenvironment in acute ischemic stroke. The prepared nanomedicine exhibited favorable biocompatibility and lesions‐targeting ability inheriting from the coated macrophage membrane. Nanoparticles could accumulate actively in the ischemic penumbra and directly protect neurons via consuming ROS and generating O_2_, which could reduce the reperfusion injury and minimize the damage caused by microcirculation dysfunction. In addition, the nanoparticles could also reverse the proinflammatory microenvironment by promoting the phenotypic transition of microglia through multiple signaling pathways, increasing the protection effects on damaged neurons. Hopefully, the application of the nanomedicine based on biomimetic cell membranes here could raise new strategy for multitarget combined treatment of stroke, as well as provide new possibilities for the treatment of brain disease.

## 4 Experimental Section

### Materials

Oleic acid (≥99%) was purchased from J&K Scientific (Beijing, China). Potassium permanganate (KMnO_4_) and ethanol was purchased from Sinopharm Chemical Reagent (Shanghai, China). FTY, methyl thiazolyl tetrazolium (MTT), and annexin V‐FITC/PI apoptosis detection kit were purchased from Dalian Meilun Biotechnology Co., Ltd (Dalian, China). Triphenyltetrazolium chloride (TTC) and 4′,6‐diamidino‐2‐phenylindole (DAPI) were purchased from Sigma‐Aldrich (St. Louis, USA). RIPA lysis solution, nuclear and cytoplasmic protein extraction kit, and BCA protein quantification kit were purchased from Beyotime (Shanghai, China). Amplite Fluorometric Hydrogen Peroxide Assay Kit was purchased from AAT Bioquest (California, USA). Rat TNF‐*α*, IL‐10 and IL‐1*β* ELISA kits were purchased from R&D Systems (Minnesota, USA).

Rabbit polyclonal antibody to NF‐*κ*B p65 (ab16502), rabbit polyclonal antibody to NF‐*κ*B p65 (phospho S536) (ab86299), rabbit polyclonal antibody to mannose receptor (ab64693), rabbit monoclonal antibody to Iba1 (ab178847), goat polyclonal antibody to Iba1 (ab5076), rabbit monoclonal antibody to NeuN (ab177487), and mouse monoclonal antibody to DNA/RNA damage (ab62623) were purchased from Abcam (Shanghai, China). Mouse monoclonal antibody to CD16/32 (ASH 1975, MA1‐7633) was purchased from ThermoFisher Scientific (Shanghai, China). Mouse monoclonal antibody to Phospho‐Stat3 (Tyr705) (M9C6, #4113) and mouse monoclonal antibody to Stat3 (124H6, #9139) were purchased from Cell Signaling Technology (Shanghai, China).

The monofilament nylon threads were purchased from Cinotech (Beijing, China).

### Cell Lines

Human neuroblastoma SH‐SY5Y cells were cultured in DMEM/F12 supplemented with 10% FBS, 100 mg mL^−1^ streptomycin, and 100 U mL^−1^ penicillin. The mouse microglia BV2 cells were cultured in DMEM containing 10% FBS, 100 mg mL^−1^ streptomycin, and 100 U mL^−1^ penicillin. The cells were grown at 37 °C in a humidified atmosphere with 5% CO_2_.

### Primary Rat Microglia Culture

Primary microglia were isolated from the cerebral cortex of 1 day old neonatal Sprague–Dawley (SD) rats. The brain tissues were dissected under aseptic conditions, and the meninges and blood vessels were carefully removed. The separated cerebral cortices were cut into pieces and digested with 0.125% trypsin (containing EDTA) for 20 min at 37 °C. The digestion was terminated with DMEM/F12 complete medium, and the mixture was dispersed evenly with Pasteur pipettes. The mixture was filtrated with a 70 µm mesh strainer before centrifugation at 800 rpm for 5 min. The collected cells were plated onto the culture dish precoated with 0.1 mg mL^−1^ poly‐d‐lysine, and cultured in DMEM/F12 complete medium. The medium was changed every 2 days until bottom astrocytes achieved complete confluence, the microglia were isolated from mixed cells by shaking at 200 rpm for 2 h. The purity of the purified microglia was investigated by flow cytometry after the cells were stained with CD11b antibody.

### Animals

Male SD rats (240–250 g) were purchased from Sippr‐BK laboratory animal Co. Ltd. (Shanghai, China). The procedures for the care of animals and all animal experiments were evaluated and approved by the Institutional Animal Care and Use Committee of the Fudan University.

Rat transient middle cerebral artery occlusion/reperfusion (tMCAO/R) model was established with a reported monofilament method.^[^ ^34^ ^]^ Rats were anesthetized with excess intraperitoneal injection of 4% sodium pentobarbital. The left common carotid artery (CCA), external carotid artery (ECA), and internal carotid artery (ICA) were exposed carefully. The CCA was then briefly ligated, and a small incision was made at the distal end of the ECA. The nylon monofilament was inserted into the ICA through the incision on ECA, the monofilament was advanced until it reached the MCA. After the blood flow to the MCA was blocked for 2 h, the monofilament was then withdrawn to allow for another 24 h reperfusion. In the sham operation group, the monofilament was immediately withdrawn after reaching the MCA.

### Preparation of Primary Macrophage Cell Membrane Vesicles

Male SD rats were injected intraperitoneally with 5 mL of 4% broth containing thioglycolate. The rats were euthanized after 3 days, and disinfected by immersing in 75% ethanol. 10 mL DMEM medium was injected intraperitoneally to extract the infiltrated macrophages. The isolated cells were allowed to adhere to the culture dish after being cultured overnight in the CO_2_ incubator. The adherent macrophages were collected and resuspended with hypotonic tris‐magnesium buffer (TM buffer containing 10 × 10^−3^ m Tris and 1 × 10^−3^ m MgCl_2_, pH 7.4). The cells were subjected to five‐cycle freezing and thawing in liquid nitrogen before being transferred to a Dounce homogenizer. The cells were then subjected to 30 passes in homogenizer before centrifugation at 1000 g for 5 min to remove the cell debris and cell nuclei. The supernatant was pooled and centrifuged at 10 000 × *g* for 20 min to remove organelles. The supernatant was then collected and centrifuged again at 100 000 × *g* for 1 h. The supernatant was discarded, and the pellet was resuspended in distilled water. The macrophage cell membrane was stored at −80 °C for further use after the protein content was quantified. The macrophage membrane dispersed in water was extruded through 400 and 200 nm polycarbonate porous membranes by an Avanti mini extruder to prepare the membrane vesicles. The size and zeta‐potential of prepared vesicles were investigated with DLS (Zetasizer Nano‐ZS, Malvern, UK).

### Preparation of MnO_2_, MnO_2_+FTY, and Ma@(MnO_2_+FTY) Nanospheres

KMnO_4_ (1.26 mmol) was fully dissolved in deionized water (100 mL) under rapid stir, then OA (6.3 mmol) was added after stirring for about 1 h. The mixture was maintained for 5 h at room temperature until the formation of brown‐black product. The products were collected with centrifugation at 14 000 rpm for 20 min, and the substrate was washed twice with distilled water and alcohol to remove the residual reactants. The purified MnO_2_ was then dispersed in distilled water and quantified with ICP‐MS, and the nanoparticles were then diluted to obtain MnO_2_ solution with a concentration of 1 mg mL^−1^. The crystallographic structure and the element composition of prepared samples were analyzed by PXRD and EDS, respectively. The specific surface area and pore size distribution of honeycomb MnO_2_ nanospheres were determined by the classic N_2_ adsorption–desorption evaluation.

For the preparation of MnO_2_+FTY nanospheres, FTY powder (20 mg) was added into MnO_2_ aqueous solution (10 mL) and ultrasonicated for 1 h to achieve a successful loading. FTY was successfully adsorbed onto the lamella of honeycomb MnO_2_ through electrostatic adsorption in this process. Excess drug was removed by centrifugation, and the amount of loaded drug was determined by high‐performance liquid chromatography (HPLC). For the preparation of macrophage‐membrane‐coated FTY‐loaded MnO_2_ nanospheres (Ma@(MnO_2_+FTY)), MnO_2_+FTY were mixed with macrophage‐membrane vesicles and coextruded through 200 nm polycarbonate porous membranes for 30 times. The size and zeta‐potential of prepared nanoparticles were investigated with DLS and NTA (NanoSight NS300, Malvern, UK), and the morphologies of prepared nanoparticles were determined with SEM and TEM.

### High‐Performance Liquid Chromatography (HPLC) Method

HPLC (Shimadzu InterSustain C18 column, 4.6 µm × 250 mm) was used to determine the loading efficiency by a UV detector at 215 nm. Mobile phase: 50% acetonitrile, 46.5% NaClO_4_ solution (0.1 m, pH 2.8), 3.5% methanol, 1 mL min^−1^, and injection volume 10 µL.

### ROS Scavenging of MnO_2_

The ROS eliminating capability of MnO_2_, MnO_2_+FTY, and Ma@(MnO_2_+FTY) nanoparticles was detected through quantifying the residual H_2_O_2_ after incubating with nanoparticles for specific times. Briefly, 40 µL MnO_2_ with different concentration (0.01, 0.02, 0.1, and 0.5 mg mL^−1^) was added into a 360 µL H_2_O_2_ (10 × 10^−6^ m) solution, and the residual H_2_O_2_ was determined with H_2_O_2_ assay kit after being incubating for 0, 1, 2, 4, and 8 h. For the comparison of catalytic capabilities among different nanoparticles, 40 µL MnO_2_, MnO_2_+FTY, and Ma@(MnO_2_+FTY) (with an equal concentration of 0.5 mg mL^−1^ MnO_2_) was added into a 360 µL H_2_O_2_ (10 × 10^−6^ m) solution, and the residual H_2_O_2_ was measured after being incubating for 1, 4 h.

### O_2_ Production Catalyzed by MnO_2_

The O_2_ producing ability by the as‐prepared MnO_2_ nanoparticles was quantitatively determined by a portable dissolved oxygen meter. Briefly, MnO_2_ nanoparticles with different concentrations (0, 6.67, and 33.33 µg mL^−1^) were incubated with 100 × 10^−6^ m H_2_O_2_ for 10 min, the content of dissolved oxygen in solution were measured per minute. The content of dissolved oxygen in distilled water was used as control.

### The In Vitro Release Profile of FTY from Ma@(MnO_2_+FTY)

In vitro release study of FTY from Ma@(MnO_2_+FTY) was performed in the condition which mimics M1 microglia. Due to the extremely poor solubility of FTY in PBS, the in vitro release experiment was carried out in sodium acetate buffer. Briefly, 500 µL Ma@(MnO_2_+FTY) nanoparticles were added in dialysis bag (MW, 3.5 kDa) and incubated in CH_3_COONa buffer (pH 7.4 or pH 5.5) with the presence of 100 × 10^−6^ m H_2_O_2_ at 37 °C. After various time points, 200 µL release medium was taken out and measured with HPLC to determine the release profile of FTY. The release profile of free FTY in CH_3_COONa buffer was used as a control.

### Cytotoxicity

The cell viability was investigated with MTT assay. For SH‐SY5Y cells, the cells were seeded into 96‐well plates with 1 ×10^4^ cells per well and cultured for 24 h. The medium was changed into DMEM/F12 complete medium containing indicated concentration of Ma@MnO_2_. For BV2 cells, BV2 cells were plated in 96‐well plates with 1 ×10^4^ cells per well and cultured for 24 h. The medium was changed into DMEM complete medium containing indicated entries of FTY, Ma@MnO_2_, and Ma@(MnO_2_+FTY) with various concentrations. After being incubating for 24 h, the cells were washed with PBS and added with 100 µL fresh medium (containing 0.5 mg mL^−1^ MTT). After incubation for another 4 h, the cells were washed with PBS and added with 100 µL DMSO to dissolve the formed formazan. The absorbance at 570 nm was recorded after shaking at 100 rpm for 10 min, and the viability of cells without any treatments was used as a control.

### Oxygen Glucose Deprivation/Reoxygenation (OGD/R) Model

The SH‐SY5Y cells were plated in 96‐well plates (1 × 10^4^ cells/well) or 24‐well plates (1 × 10^5^ cells/well) and cultured at 37 °C for 24 h. The cells were then washed with PBS, and the culture medium was then replaced with glucose‐free Hank's solution. The cells were cultured in a sealed chamber equipped with an AnaeroPack (Mitsubishi Gas Chemical, Tokyo, Japan) to maintain an anaerobic atmosphere. The cells were reoxygenated and supplemented with DMEM/F12 complete medium after OGD for 9 h.

The BV2 cells were plated in a 6‐well plate (2 × 10^5^ cells/well) or a confocal imaging dish (3 × 10^4^ cells/dish) and cultured at 37 °C for 24 h. The primary microglia were plated in 6‐well plate (2 × 10^5^ cells/well) or a confocal imaging dish (8 × 10^4^ cells/dish) and cultured at 37 °C for 24 h. The cells were then washed with PBS before the cell culture medium was changed into glucose‐free DMEM medium. The cells were cultured in anaerobic atmosphere for 4 h, then they were reoxygenated and cultured in fresh complete medium.

### ROS Scavenging Capability of Ma@MnO_2_ in OGD/R Model

To measure the ROS level in cells subjected to OGD/R, H_2_DCFDA, which would convert into the highly green fluorescent 2′,7′‐dichlorofluorescein (DCF) after being oxidized by ROS, was applied to detect the intracellular ROS formation. Briefly, SH‐SY5Y cells were plated in a 24‐well plate and treated with OGD for 9 h, the cells were then cultured in complete medium containing MnO_2_ with different concentrations for 24 h. The cells were washed with PBS before being incubating with medium containing H_2_DCFDA at concentration of 10 × 10^−6^ m for 30 min. The green signals of DCF were observed with an inverted fluorescence microscope (Leica, Wetzlar, Germany), while the fluorescence intensity of the images were quantified with ImageJ software (National Institutes of Health, Maryland, USA).

### O_2_‐Producing Capability of Ma@MnO_2_ in OGD/R Model

To determine the generation of O_2_ in SH‐SY5Y cells treated with OGD/R, [(Ru(dpp)_3_)]Cl_2_, a fluorescence probe which could be quenched by O_2_, was applied to detect the production of O_2_. Briefly, SH‐SY5Y cells were plated in a 24‐well plate (1 × 10^5^ cells/well) or a 96‐well plate and treated with OGD for 9 h, the cells were then cultured in the complete medium containing 5 × 10^−6^ m [(Ru(dpp)_3_)]Cl_2_ for 4 h. The cells were washed with PBS before being incubating with medium containing MnO_2_ with different concentrations for the indicated times. After 0.5 h, the fluorescence intensity(*λ* = 620 nm)of cells in the 96‐well plate was analyzed with a microplate reader. After 12 h, the fluorescence intensity of the cells in the 24‐well plate was analyzed with flow cytometry. For the cells treated with OGD/R and different formulations, the red signals of [(Ru(dpp)_3_)]Cl_2_ were observed with an inverted fluorescence microscope.

### Neuroprotective Effects of Ma@MnO_2_ in OGD/R Model

The neuroprotective effects of Ma@MnO_2_ were determined with the cell viability assay. Briefly, SH‐SY5Y cells were plated in a 96‐well plate and treated with OGD for 9 h, the cells were then cultured in the complete medium containing Ma@MnO_2_ with different concentrations for 24 h. The cell viability was then investigated with the MTT assay. For the investigation of antiapoptotic effect of Ma@(MnO_2_+FTY), SH‐SY5Y cells were plated in a 12‐well plate and treated with OGD for 9 h, and the cells were then treated with different formulations for 24 h. Cell apoptosis was detected by staining with annexin V‐FITC and propidium iodide, and analyzed by flow cytometry (Beckman, Indianapolis, USA).

### ROS Scavenging Capability of Ma@(MnO_2_+FTY) in BV2 Cells OGD/R Model

Briefly, BV2 cells were plated in a 24‐well plate and treated with OGD for 4 h, the cells were then cultured in complete medium containing different formulations for 24 h. The cells were washed with PBS before being incubating with medium containing H_2_DCFDA at concentration of 10 × 10^−6^ m for 30 min. The green signals of DCF were observed with an inverted fluorescence microscope.

### The Internalization Mechanism Study of Ma@(MnO_2_+FTY) in BV2 Cells

Briefly, BV2 cells were plated in a 12‐well plate (1 × 10^5^ cells/well) and cultured for 24 h. The cells were then cultured at 4 °C or pretreated with different endocytosis inhibitors at 37 °C for 1 h, including filipin (inhibiting caveolin‐mediated endocytosis, 5 µg mL^−1^), chlorpromazine (CPM, inhibiting clathrin‐mediated endocytosis, 5 µg mL^−1^), and wortmannin (WTM, inhibiting micropinocytosis, 1 µg mL^−1^). Then, the medium was removed and the cells were washed with PBS for 3 times. The cells were incubated with DID‐labeled Ma@(MnO_2_+FTY) nanoparticles for 1 h, after washing with PBS, the cells were collected and the fluorescence intensity was detected with flow cytometry.

### Western Blot

Immunoblotting analysis was chosen to detect the expression of specific proteins. Microglia were plated in a 6‐well plate and treated with OGD for 4 h, and incubated with different formulations (FTY was normalized to 0.36 × 10^−6^ m, while MnO_2_ was normalized to 3.13 µg mL^−1^) for 24 h after OGD. The cells were lysed with RIPA lysis buffer and the protein concentration was determined with BCA protein assay. To detect the translocation of P65, the nuclear and cytoplasmic proteins were isolated with an extraction kit. To investigate the expression of *p*‐P65 in the ischemic hemisphere, the isolated brain tissues were homogenated and lysed with RIPA lysis buffer. A total of 20 µg sample was resolved by 10% SDS‐PAGE for the detection of STAT3 related proteins, 10 µg proteins were separated by 12% SDS‐PAGE for the detection of NF‐*κ*B related proteins. The proteins were transferred to polyvinylidene fluoride membranes. The membranes were then blocked with 5% nonfat dry milk and incubated with anti‐*β*‐actin (1:1000), antihistone (1:1000), anti‐P65 (1:2000), anti‐p‐P65 (1:2000), anti‐STAT3 (1:1000), and anti‐p‐STAT3 (1:1500) at 4 °C overnight. The membranes were incubated with HRP‐conjugated secondary antibody (1:1000) for 1 h. The blots were detected with electrochemiluminescent HRP substrate and analyzed with Image Lab software (Bio‐Rad).

### Immunofluorescent Staining

Immunofluorescent staining was carried out to detect the expression level of CD16/32 (M1 marker), CD206 (M2 marker) and p‐P65 in microglia. The primary microglia were plated in confocal imaging dish (8 × 10^4^/dish) and treated with OGD/R. The cells were incubated with different formulations (FTY was normalized to 0.36 × 10^−6^ m, MnO_2_ was normalized to 3.13 µg mL^−1^) for 24 h after OGD. The cells were fixed in 4% paraformaldehyde for 15 min, and washed with cold PBS twice. The cells were then permeabilized in 0.3% Tween‐20 for 20 min, and blocked by 1% BSA for 30 min. Cells were incubated with primary antibody anti‐CD16/32 (1:100), anti‐CD206 (1:200), and anti‐p‐P65 (1:500) at 4 °C overnight. After washing with PBS for 3 times, the fixed cells were incubated with secondary antibody goat antirabbit IgG Alexa Flour 488 (1:1000) and goat antimouse IgG Alexa Flour 555 (1:1000). The cells were observed with confocal laser scanning microscope (Carl Zeiss LSM710, Oberkochen, Germany) after stained with DAPI, and the fluorescence intensity of the images was quantified with ImageJ software. Primary antibodies including anti‐CD206 (1:200), anti‐p‐P65 (1:50), anti‐NeuN (1:1000), anti‐8‐OHG (1:1000), anti‐Iba‐1 (1:100, rabbit), anti‐Iba‐1 (1:200, goat), and anti‐iNOS (1:50) and secondary antibody including goat antimouse IgG Alexa Flour 555 (1:500), donkey antirabbit IgG Alexa Flour 647 (1:500), donkey antigoat IgG Alexa Flour 488 (1:1000), and goat antirabbit IgG Alexa Flour 488 (1:1000) were employed for the immunofluorescence staining of frozen brain sections.

### Targeting Capability of Ma@(MnO_2_+FTY) Nanoparticles In Vivo

After 2 h of reperfusion, tMCAO/R rats were injected intravenously with free fluorescence probe (WGA conjugated with Alexa Fluor 633) and Ma@(MnO_2_+FTY) nanoparticles labeled with above probe. The rats were sacrificed and the brains were removed at 24 h post reperfusion. The isolated brains were imaged by In Vivo Imaging System (IVIS) (Caliper). The brains were sliced into 2 mm width coronal sections and imaged with IVIS again.

### The Tissue Distribution Experiment

Briefly, the tMCAO/R rats were injected intravenously with Ma@(MnO_2_+FTY) nanoparticles (the dose of MnO_2_ were normalized to 3 mg kg^−1^). Then the rats were euthanized at 24 h post reperfusion, the heart, liver, spleen, lung, kidney, and brain were removed for the following detection. After the programmatic digestion with concentrated nitric acid, the content of Mn in organs was measured by ICP‐MS. In order to maintain the consistency of particle size and zeta‐potential, erythrocyte membrane‐coated FTY‐loaded MnO_2_ nanoparticles (RM@(MnO_2_+FTY)) were applied as a control.

### Neuroprotection Effect Evaluated with TTC

The tMCAO/R rats were randomly divided into five groups after 2 h of reperfusion and injected intravenously with saline (MCAO), 1.5 mg kg^−1^ FTY (FTY‐Low), 3 mg kg^−1^ FTY (FTY‐High), and Ma@MnO_2_ and Ma@(MnO_2_+FTY) (with an equal dose of 3 mg kg^−1^ MnO_2_), and the dose of FTY in Ma@(MnO_2_+FTY) group was 1.5 mg kg^−1^. The infarct area was monitored by MRI at 24 h post reperfusion. The rats were then sacrificed, the removed brains were frozen at −20 °C and sliced into 2 mm width coronal sections. The brain sections were next stained with 2% TTC in PBS buffer and imaged with camera. The infarct area (white part) the images were quantified with ImageJ software.

### MRI Imaging In Vivo

The tMCAO/R rats with different treatment were anaesthetized with isoflurane. To monitor the infarct area, T2‐weighted coronal images of the brain were recorded with 7 T MR scanner for small animal imaging system (BioSpec70/20USR, Burke, Germany) at 24 h post reperfusion. The acquisition parameters for T2‐weighted MRI imaging: TR  =  2000.0 ms, TE  =  25.0 ms, flip angel = 90.0°, NEX  =  3, FOV  =  3.00 cm, matrix  =  128, section thickness  =  1.00 mm, scan  =  15, and echo = 1/1.

### Neuroscore Assessment

After the model rats were treated with different formulations, neuroscore assessment was performed using a well‐established five‐point scale method (rating scale: 4 = spontaneous circling; 3 = circling to left by pulling the tail; 2 = decreased grip strength of left forepaw; 1 = failure to extent left forepaw; and 0 = no deficit).^[^ ^34^ ^]^

### Investigation of the Pharmacokinetics of Ma@(MnO_2_+FTY) Nanoparticles in Rats

Rats were injected intravenously with Ma@MnO_2_, Ma@(MnO_2_+FTY) nanoparticles and free FTY (the dose of FTY were normalized to 1.5 mg kg^−1^, the dose of MnO_2_ were normalized to 3 mg kg^−1^), blood samples were collected through the eyeball at scheduled time points. For the measurement of Mn^2+^, the blood samples were fully digested in the concentrated nitric acid, and the content of Mn^2+^ was further analyzed with ICP‐MS.

The measurement of FTY was carried out according to a reported method.^[^ ^35^ ^]^ For the extraction of FTY from blood samples, 500 µL of sodium hydroxide solution (0.1 N) was added into 200 µL of blood samples before adding with 6 mL of dichloromethane and *tert*‐butyl‐methylether (25:75). After a vortex for 45 min, the mixtures were centrifuged at 2000 × *g* for 10 min. The organic phase was collected and dried under N_2_ before being redissolved in a 150 µL mobile phase. The mixtures were sonicated for 10 min and centrifuged at 15 000 × *g* for 5 min before being injected into LC‐MS/MS (parent molecular ion of 308.3, and daughter ion of 255.3). The column used for HPLC was a 4.6 µm × 250 mm C18 column (Shimadzu InterSustain, Kyoto, Japan). The samples were eluted with 90% methanol and 10% ammonium acetate solution (10 × 10^−3^ m) at 0.9 mL min^−1^, and the temperature was maintained at 40 °C.

### The Polarization of Activated Microglia from M1 to M2 In Vivo

Briefly, model rats were sacrificed at 24 h post reperfusion, and the brain was isolated and chopped for the preparation of single cell suspension. After ground and filtrated on a 70 µm mesh strainer, the mixture was centrifuged at 1300 rpm for 10 min. The pellet was then redispersed in 30% percoll and loaded on 70% percoll for density gradient centrifugation. After centrifugation for 30 minutes at 500 × *g*, cells were obtained from the intermediate phase and stained for flow cytometry analysis. Antibodies including CD11b monoclonal antibody (0.5 µg mL^−1^, APC‐eFluor 780, eBioscience), CD45 monoclonal antibody (0.4 µg mL^−1^, APC, eBioscience), CD16/32 monoclonal antibody (0.3 µg mL^−1^, PE, eBioscience), CD206 monoclonal antibody (0.6 µg mL^−1^, PE‐Cyanine7, eBioscience) were used to identify specific cells. The microglia were presented as CD45^int^CD11b^+^ cells; among microglia, M1 microglia were presented as CD16/32^+^ cells, and M2 microglia were presented as CD206^+^ cells.

### Hemolysis Test

Red blood cells (RBCs) isolated from rat blood were used for the hemolysis evaluation. The RBCs were diluted to 1/10 of their volume with isotonic PBS working solution. Then, 0.2 mL RBC suspension was added into the following samples (0.8 mL): MnO_2_, MnO_2_+FTY, Ma@MnO_2_ and Ma@(MnO_2_+FTY) nanoparticles at concentrations ranging from 3.125 to 200 µg mL^−1^. RBCs added into PBS were used as a negative control, and RBCs added into the distilled water were used as a positive control. The mixtures were then gently shaken (50 rpm) for 1 h at 37 °C before centrifugation at 3000 rpm for 10 min. The supernatants were collected and the absorbance at 541 nm was detected on a microplate reader. The hemolysis percentages of samples were calculated with the following equation: hemolysis percentage = ((absorbance of sample − absorbance of negative control)/(absorbance of positive control − absorbance of negative control)) × 100%.

### Measurement of Cytokines in Ischemic Hemisphere

To investigate the expression of inflammation‐related cytokines in the ischemic hemisphere, the isolated brain tissues were homogenated and lysed with RIPA lysis buffer. The content of TNF‐*α*, IL‐10, and IL‐1*β* in the samples were determined with the ELISA kit according to the protocol.

### Hematoxylin–Eosin (H&E) Staining

For the safety evaluation, tMCAO/R rats were sacrificed after being treated with different formulations for 24 h, and main organs were excised and fixed in 4% paraformaldehyde. The tissue sections embedded in paraffin were stained with hematoxylin and eosin, and observed with a microscope. Tissue sections of sham group were used as a control.

### Statistical Analysis

All statistical analysis were performed with GraphPad Prism software (8.0), and all results were reported as means ± standard deviation (SD). The statistical significance between groups were studied by unpaired *t*‐test, which was presented as ^*^ *P* < 0.05, ^**^ *P* < 0.01, and ^***^ *P* < 0.001.

## Conflict of Interest

The authors declare no conflict of interest.

## Supporting information

Supporting Information

## Data Availability

Research data are not shared.
