# Supplementary material for: Macrophage‐Disguised Manganese Dioxide Nanoparticles for Neuroprotection by Reducing Oxidative Stress and Modulating Inflammatory Microenvironment in Acute Ischemic Stroke
Source: Adv Sci (Weinh). 2021 Aug 26;8(20):2101526. doi: 10.1002/advs.202101526 (PMC8529435; doi:10.1002/advs.202101526)
Supplement: Supplementary file 1 — Supporting Information [file ADVS-8-2101526-s001.pdf]

© 2020 Wiley-VCH GmbH

ADVANCED SCIENCE
Open Access

Supporting Information

for *Adv. Sci.,* DOI: 10.1002/advs.202101526

Macrophage-Disguised Manganese Dioxide Nanoparticles for Neuroprotection by Reducing Oxidative Stress and Modulating Inflammatory Microenvironment in Acute Ischemic Stroke

*Chao Li, Zhenhao Zhao, Yifan Luo, Tingting Ning, Peixin Liu, Qinjun Chen, Yongchao Chu, Qin Guo, Yiwen Zhang, Wenxi Zhou, Hongyi Chen, Zheng Zhou, Yu Wang, Boyu Su, Haoyu You, Tongyu Zhang, Xuwen Li, Haolin Song, Chufeng Li, Tao Sun, Chen Jiang**

Supporting Information

Macrophage-Disguised Manganese Dioxide Nanoparticles for Neuroprotection by Reducing Oxidative Stress and Modulating Inflammatory Microenvironment in Acute Ischemic Stroke

*Chao Li, Zhenhao Zhao, Yifan Luo, Tingting Ning, Peixin Liu, Qinjun Chen, Yongchao Chu, Qin Guo, Yiwen Zhang, Wenxi Zhou, Hongyi Chen, Zheng Zhou, Yu Wang, Boyu Su, Haoyu You, Tongyu Zhang, Xuwen Li, Haolin Song, Chufeng Li, Tao Sun, Chen Jiang**

C. Li, Z. Zhao, Y. Luo, T Ning, P. Liu, Q. Chen, Y. Chu, Q. Guo, Y. Zhang, W. Zhou, H. Chen, Z. Zhou, Y. Wang, B. Su, H. You, T. Zhang, X. Li, H. Song, C. Li, Prof. T. Sun, Prof. C. Jiang

Key Laboratory of Smart Drug Delivery (Ministry of Education)
State Key Laboratory of Medical Neurobiology and MOE Frontiers Center for Brain Science
Department of Pharmaceutics
School of Pharmacy
Research Center on Aging and Medicine
Fudan University
Shanghai 201203, China
E-mail: jiangchen@shmu.edu.cn

Contents:

Figure S1. UV-vis absorption spectra of prepared nanoparticles

Figure S2. PXRD pattern of MnO_2 nanospheres

Figure S3. EDS analysis of MnO_2 nanospheres

Figure S4. SEM images of MnO_2 nanospheres

Figure S5. Nitrogen sorption isotherms of MnO_2 nanospheres

Figure S6. The pore distribution of MnO_2 nanospheres

Figure S7. The change of zeta-potential values of optimal prescription screening for

the ratio of cell membrane to MnO_2+FTY

Figure S8. The NTA result of Ma@(MnO_2+FTY)

Figure S9. Membrane surficial proteins on Ma@(MnO_2+FTY) nanoparticles

Figure S10. Representative TEM images of immunostaining of CD11b protein on the Ma@(MnO_2+FTY) nanoparticles

Figure S11. The stability study of Ma@(MnO_2+FTY)

Figure S12. The production of O_2 catalyzed by MnO_2

Figure S13. The disintegration of Ma@(MnO_2+FTY) in PBS 6.0 containing 100 μM H_2O_2

Figure S14. T1-weighted MR images of Ma@(MnO_2+FTY)

Figure S15. The biocompatibility investigation of Ma@MnO_2 on SH-SY5Y cells

Figure S16. The fluorescence images of ROS in SH-SY5Y cells

Figure S17. The generation of O_2 in SH-SY5Y cells with different treatment

Figure S18. The safety of nanomedicine on BV2 cells

Figure S19. The uptake inhibition experiment of Ma@(MnO_2+FTY) in BV2 cells

Figure S20. The cellular uptake of Ma@(MnO_2+FTY) in BV2 cells

Figure S21. The fluorescence images of ROS in BV2 cells

Figure S22. The purity identification of isolated primary microglia

Figure S23. The bio-distribution of Ma@(MnO_2+FTY) in main organs of tMCAO/R rats

Figure S24. T1-weighted MR images of tMCAO/R rat

Figure S25. The polarization of activated microglia from M1 to M2 *in vivo*

Figure S26. The fluorescence images of p-P65 in microglia in ischemic hemisphere

Figure S27. The protein expression of p-P65 in the ischemic hemisphere

Figure S28. The neuroscore assessment of tMCAO/R rats treated with different formulations

Figure S29. The hemocompatibility study of Ma@(MnO_2+FTY) by hemolysis test

Figure S30. The liver function study

Figure S31. The H&E staining images of main organs with full view

Figure S32. Representative images of main organs with H&E staining

Table S1. Pharmacokinetic parameters of free FTY and Ma@(MnO_2+FTY) nanoparticles

Supplementary Figures

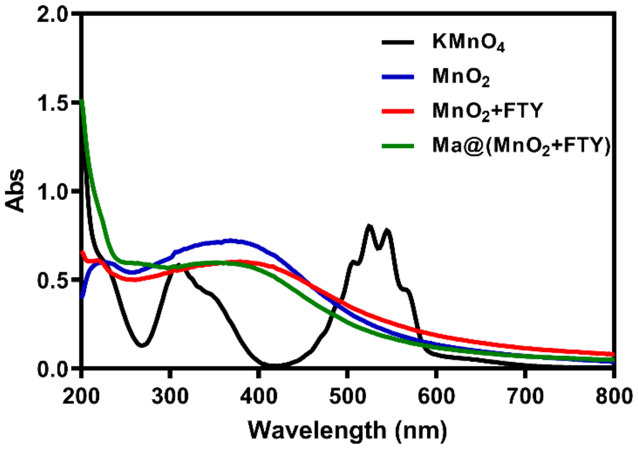

Figure S1. UV-vis absorption spectra of $KMnO_4$ solution, MnO_2, FTY-loaded MnO_2, and Ma@(MnO_2+FTY).

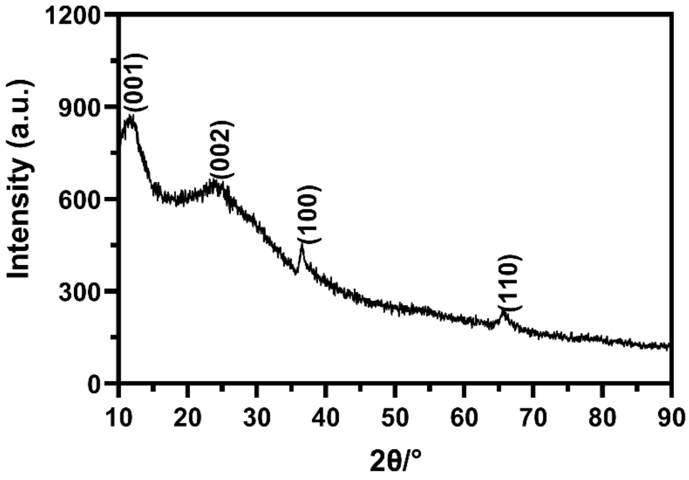

Figure S2. PXRD pattern of MnO_2 nanospheres.

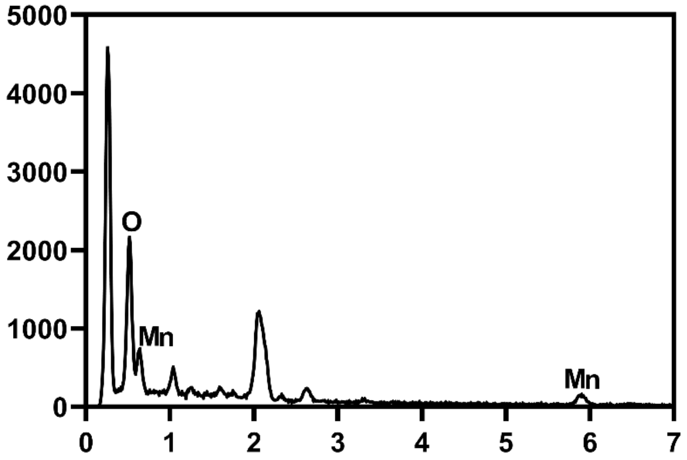

Figure S3. EDS analysis of MnO_2 nanospheres.

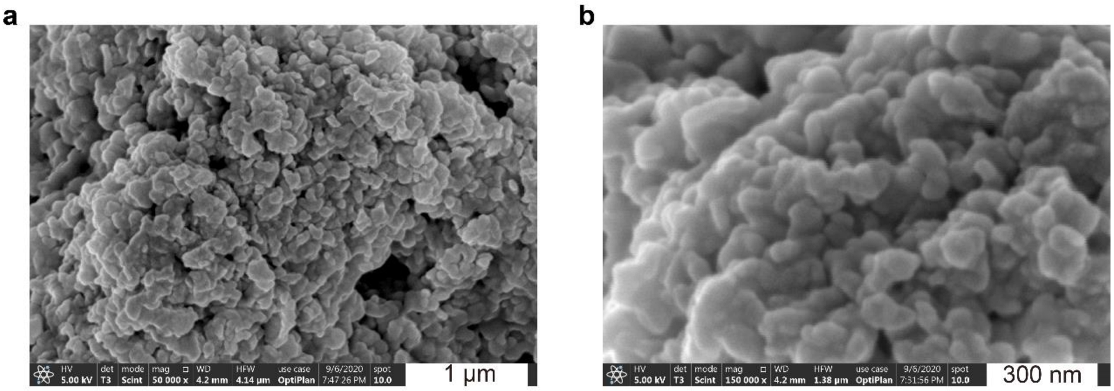

Figure S4. SEM images of MnO_2 nanospheres.

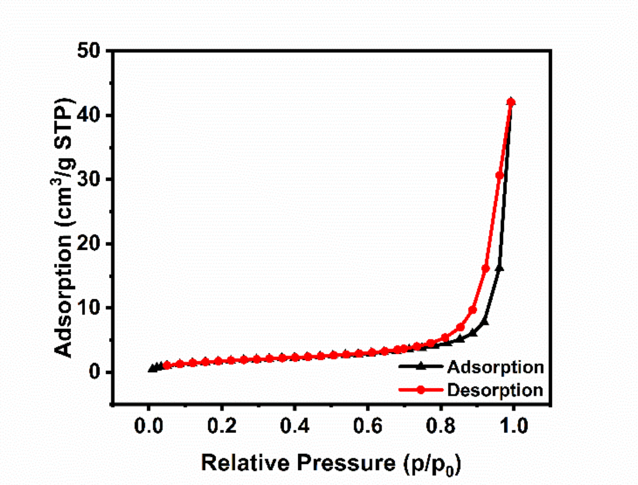

Figure S5. Nitrogen sorption isotherms of MnO_2 nanospheres.

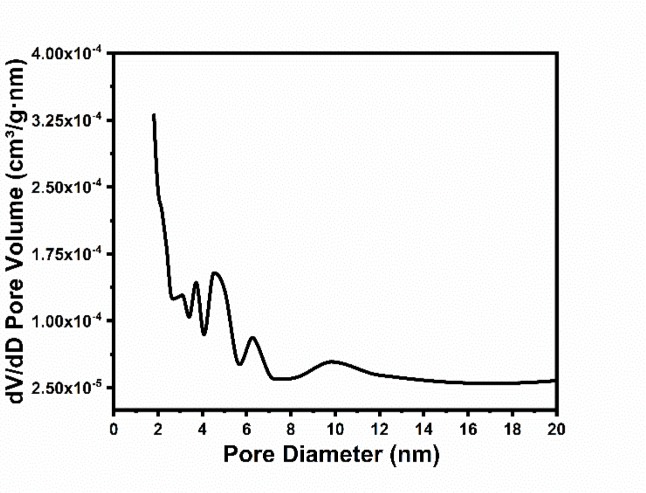

Figure S6. The pore distribution of MnO_2 nanospheres.

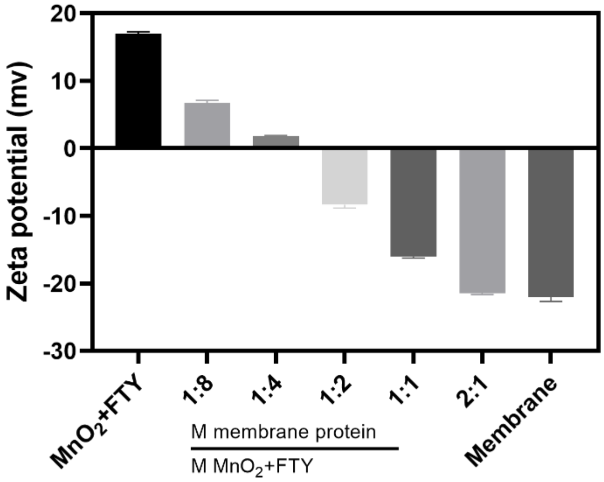

Figure S7. Optimal prescription screening for the ratio of cell membrane (quantified by protein content) to MnO_2+FTY by monitoring the change of zeta-potential values. Data are reported as means ± SD, n = 3.

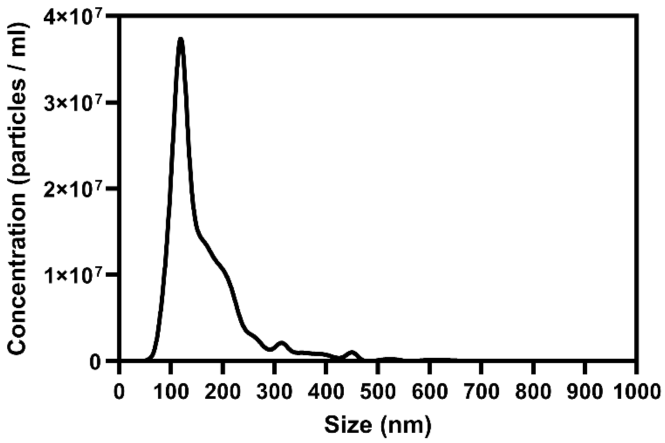

Figure S8. The NTA result of size distribution and the corresponding concentration of Ma@(MnO_2+FTY) nanoparticles.

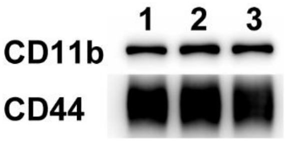

Figure S9. Membrane surficial proteins in macrophage membrane (1), macrophage membrane vesicles (2) and Ma@(MnO_2+FTY) nanoparticles (3), analyzed with western-blots.

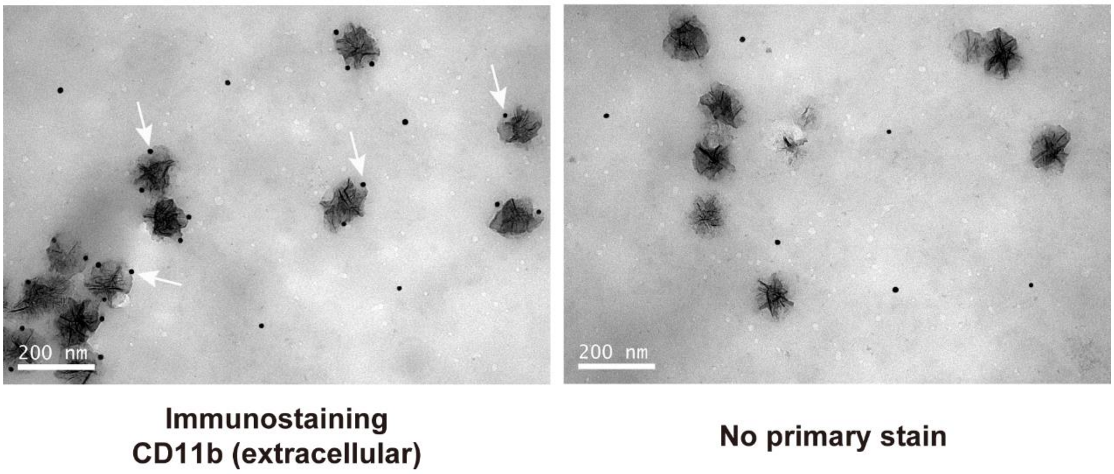

Figure S10. Representative TEM images of immunostaining which revealed the right-out-side CD11b (extracellular domain) on the Ma@(MnO_2+FTY) nanoparticles.

(Scale bar, 200 nm).

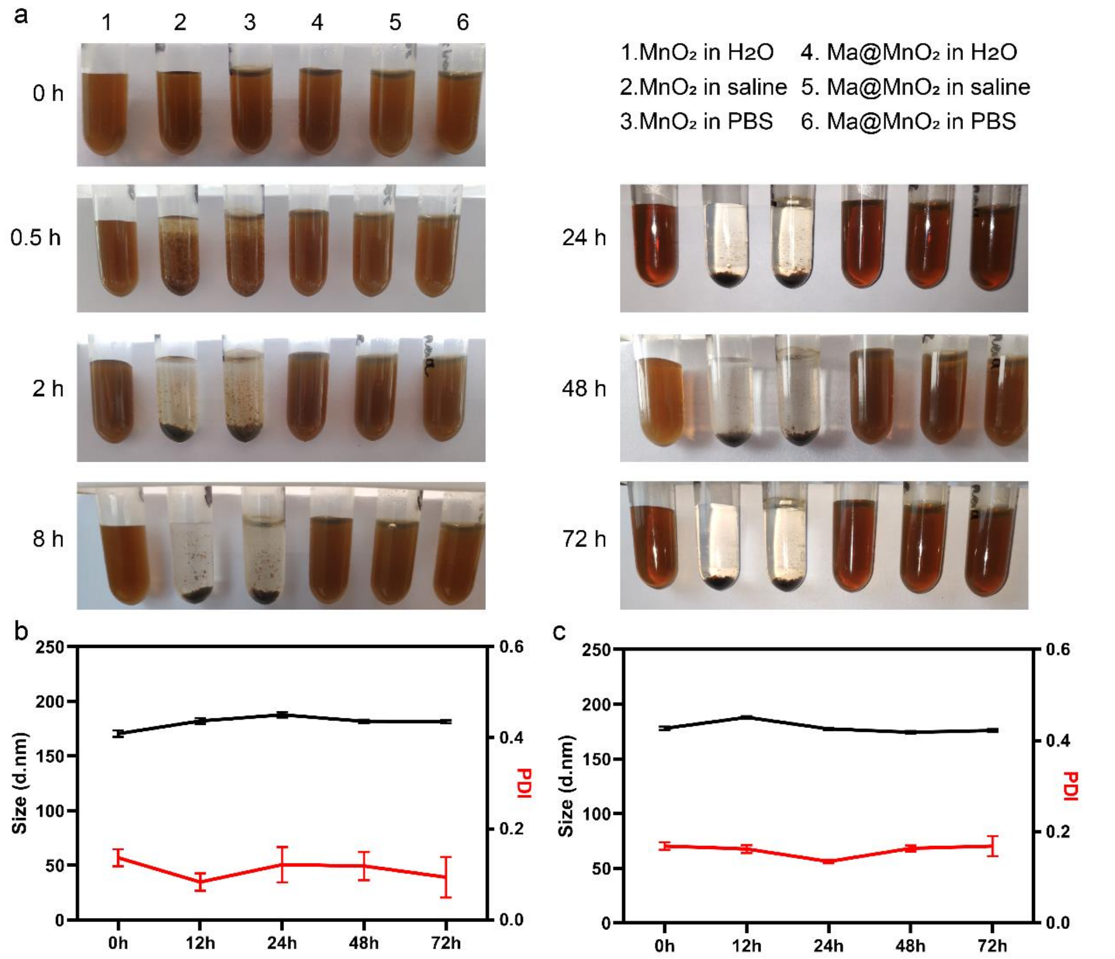

Figure S11. (a) Representative images of MnO_2 and Ma@MnO_2 in H_2O, saline and PBS over 72 h. The size and PDI of Ma@MnO_2 (b) and Ma@(MnO_2+FTY) (c) in PBS over 3 days. Results are presented as means ± SD, n = 3).

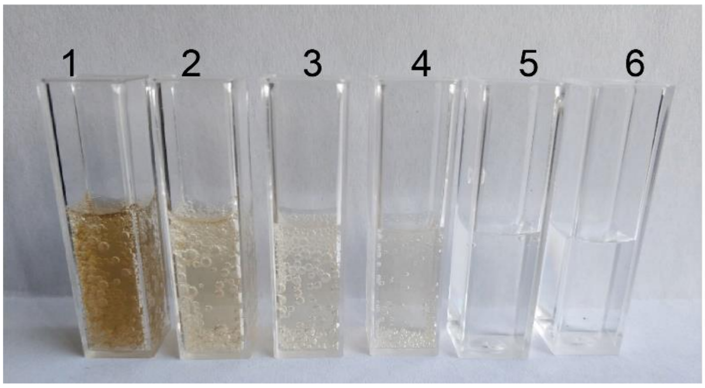

Figure S12. The production of O_2 after adding different concentration of MnO_2 into H_2O_2 (100 mM) solution for 5 min. The concentrations of MnO_2 from left to right are 100, 50, 25, 12.5, 6.25 and 0 μg mL^{-1}, respectively.

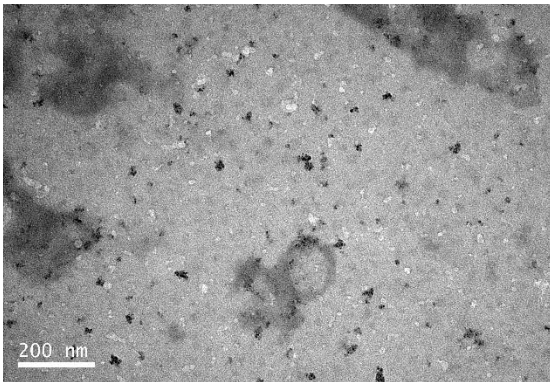

Figure S13. Representative TEM images of Ma@(MnO_2+FTY) after incubation in PBS 6.0 with 100 μM H_2O_2 at 37 °C for 30 min.

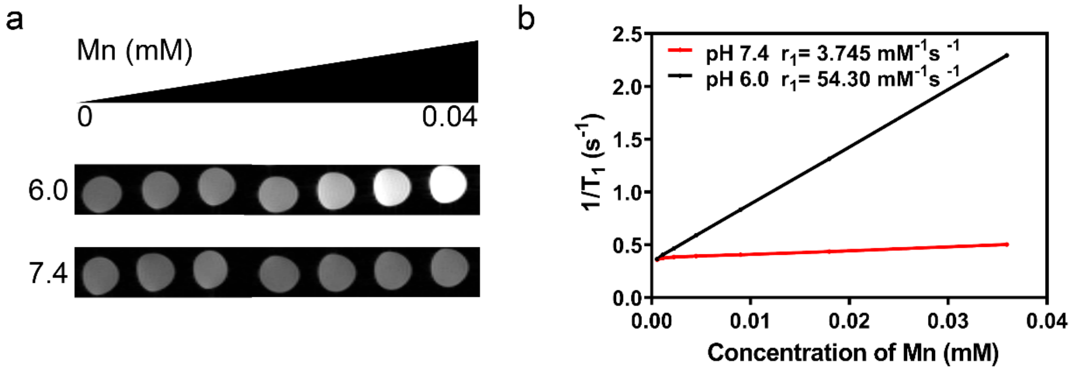

Figure S7. (a) T1-weighted MR images of Ma@(MnO_2+FTY) after incubation in PBS 6.0 and 7.4, all with 100 μM H_2O_2. (b) T1 relaxation rate (1/T1) raised linearly with the concentration of Mn^{2+} in Ma@(MnO_2+FTY) nanoparticles processed with 100 μM H_2O_2 in PBS 6.0, r1 were 54.30 mM^{-1} s^{-1} and 3.745 mM^{-1} s^{-1} for Ma@(MnO_2+FTY) at pH 6.0 and 7.4, respectively.

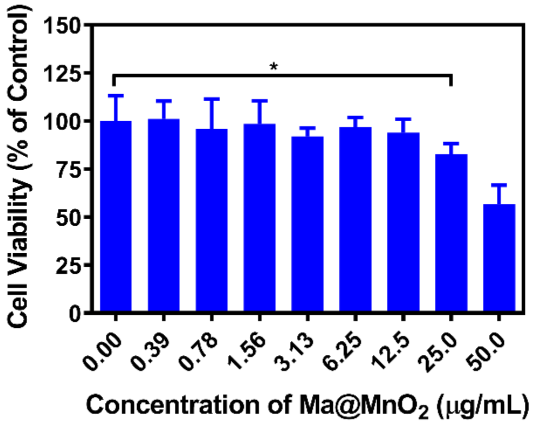

Figure S15. The biocompatibility investigation of Ma@MnO_2 on SH-SY5Y cells, cells were incubated with Ma@MnO_2 in different concentration for 1 day. Results are presented as means ± SD, n = 5, $*P < 0.05$.

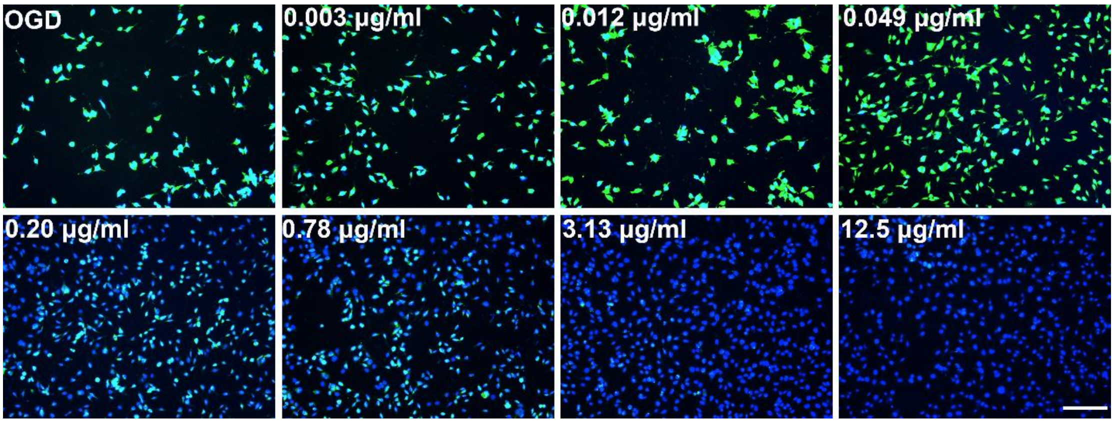

Figure S16. Representative images of the H_2D-CFDA fluorescence in SH-SY5Y cells treated with OGD/R or different concentration of Ma@MnO_2 (Scale bar, 100 μm).

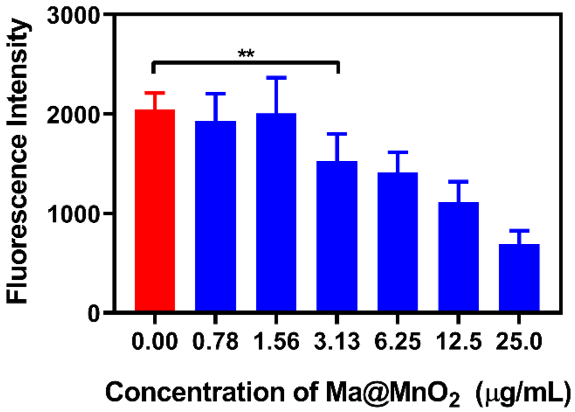

Figure S17. The generation of O_2 in SH-SY5Y cells treated with OGD/R, cells in 96-well plates were incubated with Ma@MnO_2 of different concentration for 30 min, and the fluorescence intensity at 620 nm was analyzed with a microplate reader. Data are reported as means ± SD, n = 6, $^{**}P < 0.01$.

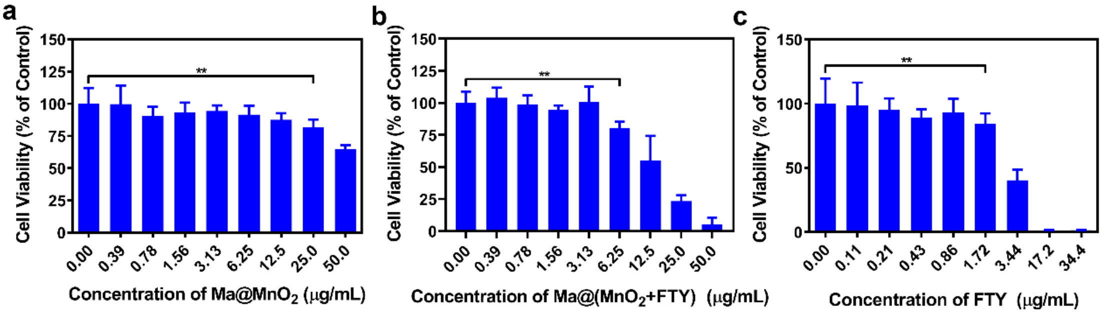

Figure S18. The safety of Ma@MnO_2, Ma@(MnO_2+FTY), and FTY on BV2 cells, cells were incubated with drugs in different concentration for 24 hours. Results are presented as means ± SD, n = 5, **$P < 0.01$.

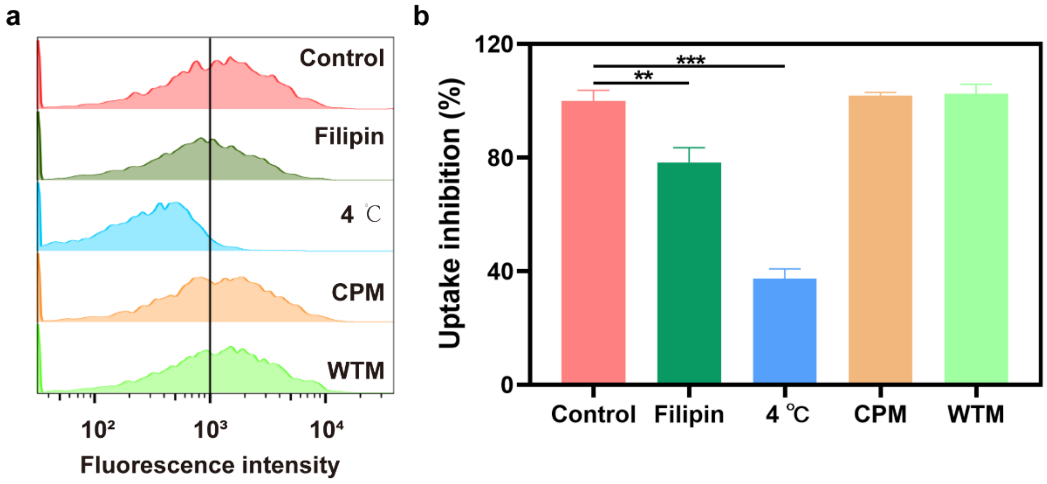

Figure S19. The internalization mechanism of Ma@(MnO_2+FTY) in BV2 cells. a) The representative flow cytometry analysis results of the fluorescence intensity in BV2 cells with different treatment. b) The semi-quantitative results of the flow cytometry analysis. Data are presented as means ± SD, n = 3, **$P < 0.01$, ***$P < 0.001$.

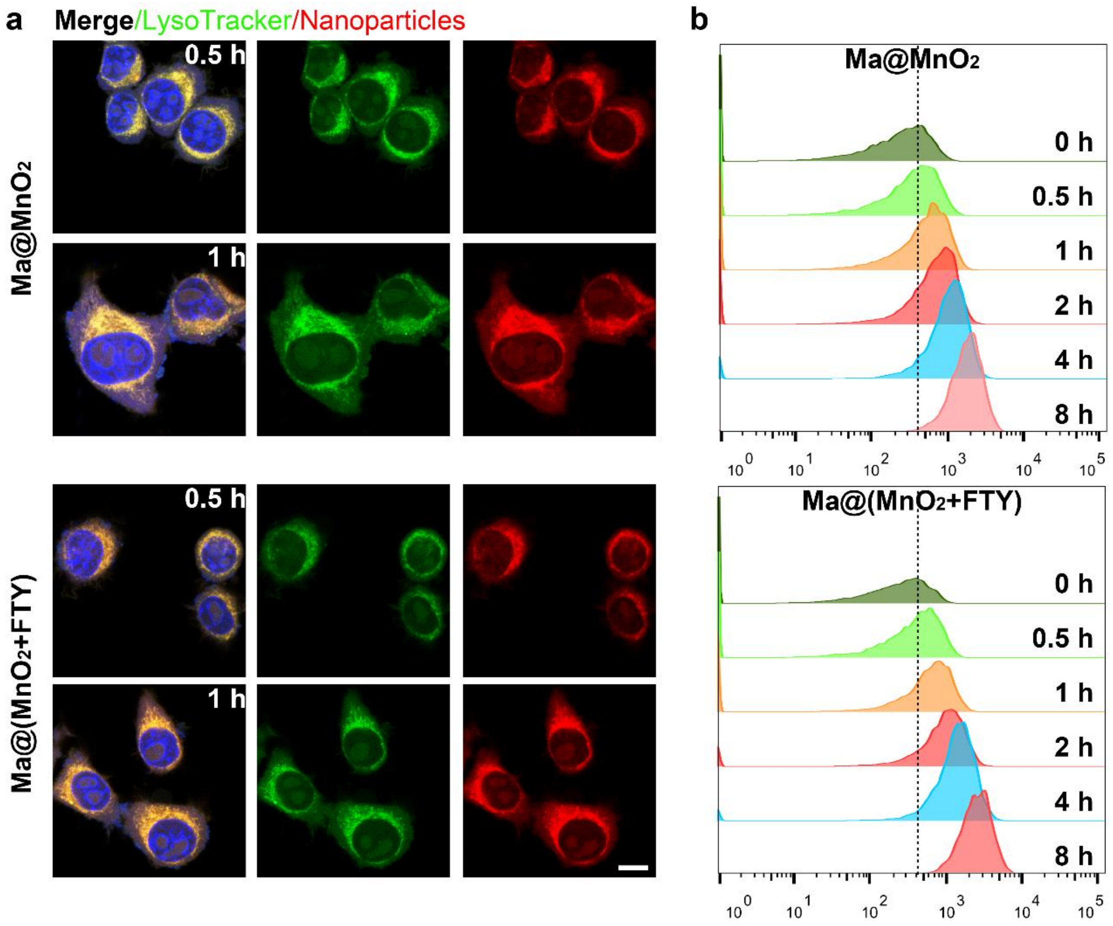

Figure S20. (a) Representative images of cellular uptake in BV2 cells after incubated with Ma@MnO_2 or Ma@(MnO_2+FTY) for 0.5 h, 1 h, (Scale bar, 100 μm). (b) The flow cytometry results of cellular uptake, treated with above nanoparticles for different time.

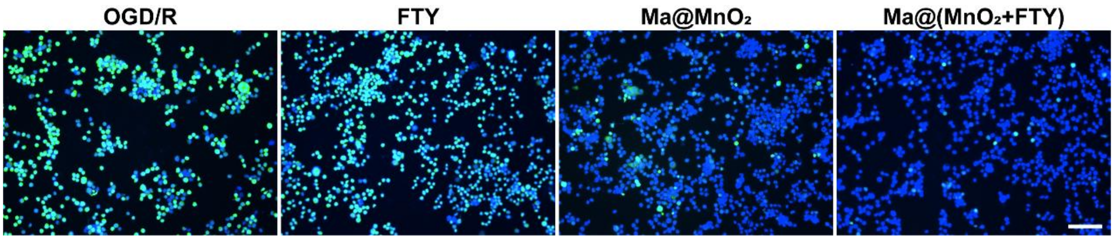

Figure S21. Representative images of the H_2D-CFDA fluorescence in BV2 cells treated with OGD/R or different formulations (Scale bar, 100 μm).

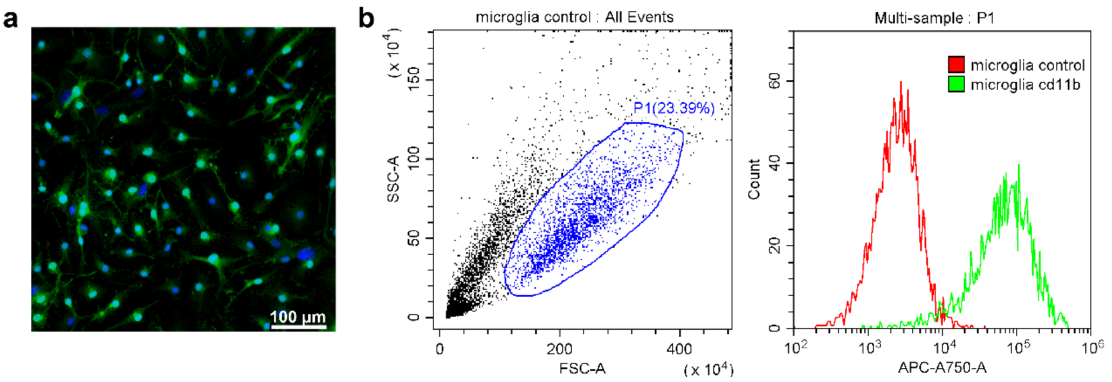

Figure S22. (a) Representative immunofluorescence images of primary microglia stained with CD11b (Scale bar, 100 μm). (b) The flow cytometry results of primary microglia stained with CD11b, confirming cell purity greater than 97%.

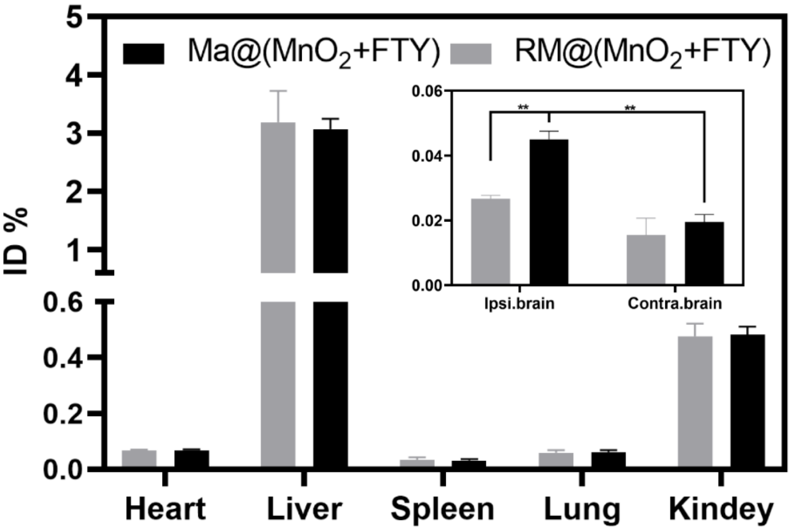

Figure S23. The bio-distribution of Ma@(MnO_2+FTY) in main organs of tMCAO/R

rats. The ID % of RM@(MnO_2+FTY) nanoparticles were applied as a control. Data are presented as means ± SD, n = 3, **$P < 0.01$.

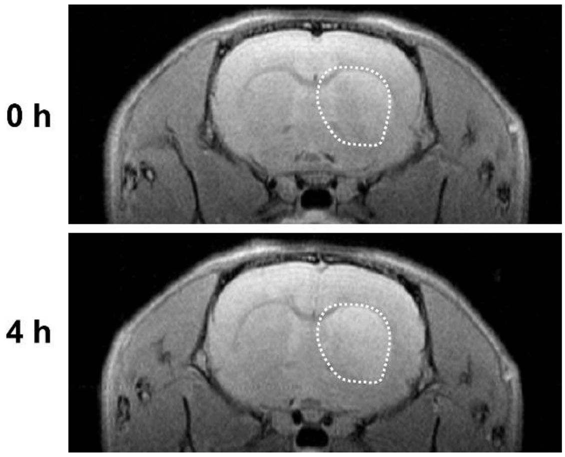

Figure S24. T1-weighted MR images of tMCAO/R rat after injection with Ma@(MnO_2+FTY) for 4 h.

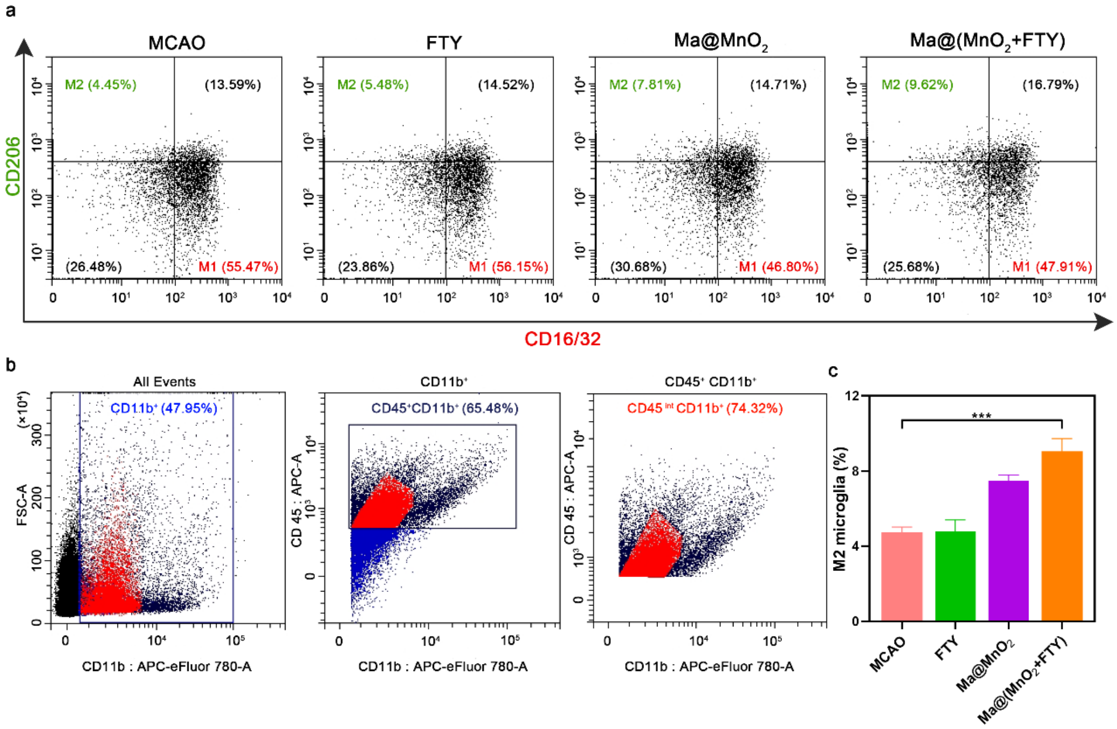

Figure S25. Treated with Ma@(MnO_2+FTY) promoted the polarization of activated microglia from M1 to M2 in ischemic brain. a) Representative images of microglia

phenotype polarization analyzed with flow cytometry. b) The gating strategy of $CD45^{int}CD11b^{+}$ microglia. c) The semi-quantitative results of the flow cytometry analysis. Data are reported as means ± SD, n = 3, ***$P < 0.001$.

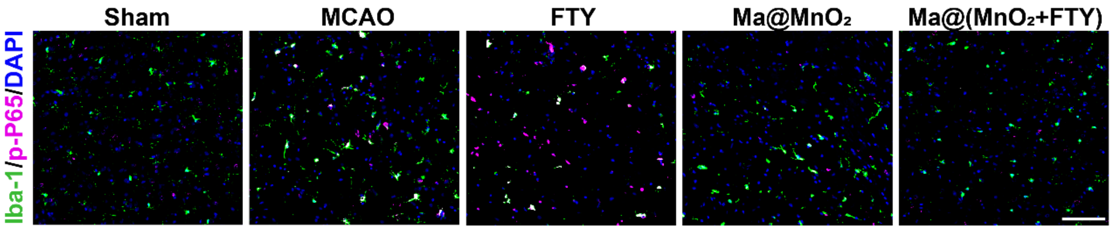

Figure S26. Representative images of the expression of p-P65 in microglia in ischemic hemisphere (Scale bar, 100 μm).

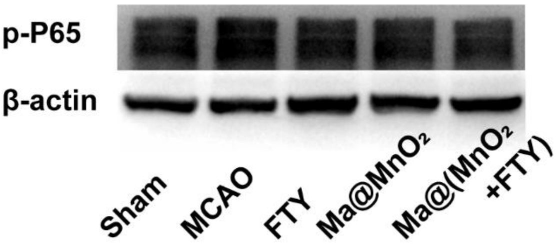

Figure S27. Expression of p-P65 in the ischemic hemisphere of MCAO rats after treated with different drugs.

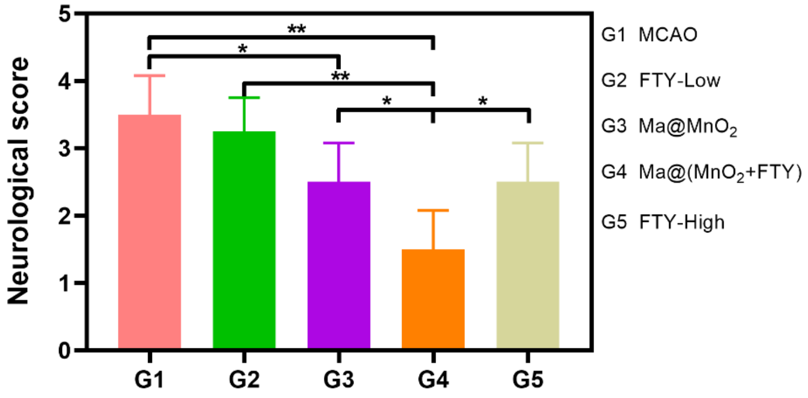

Figure S28. The neurological assessment of tMCAO/R rats treated with different formulations. Data are presented as means ± SD, n = 3, *$P < 0.01$, **$P < 0.01$.

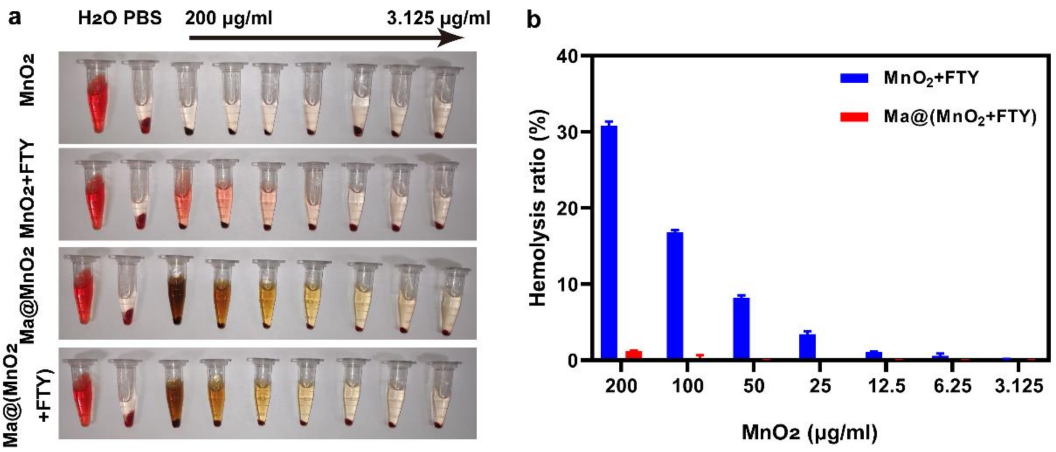

Figure S29. Representative images of hemolysis experiment with different formulations. Data are presented as means ± SD, n = 3.

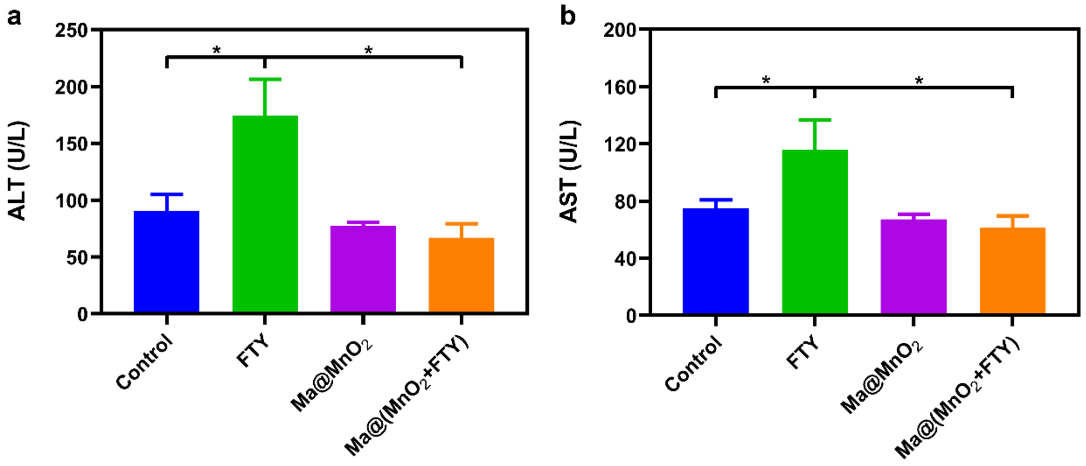

Figure S30. The level of AST and ALT in rats after treated with different formulations. Results are reported as means ± SD, n = 3, *$P < 0.05$.

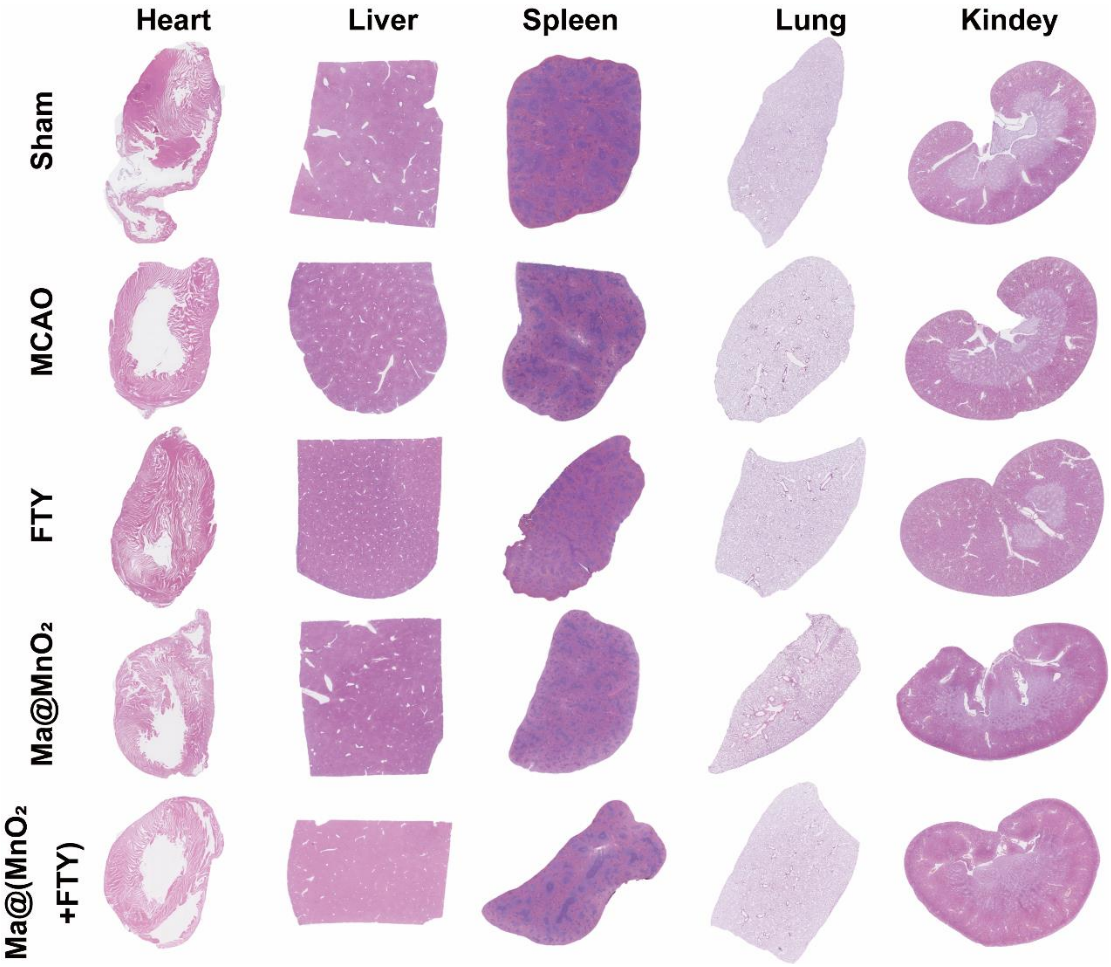

Figure S31. The H&E staining images of main organs with full view.

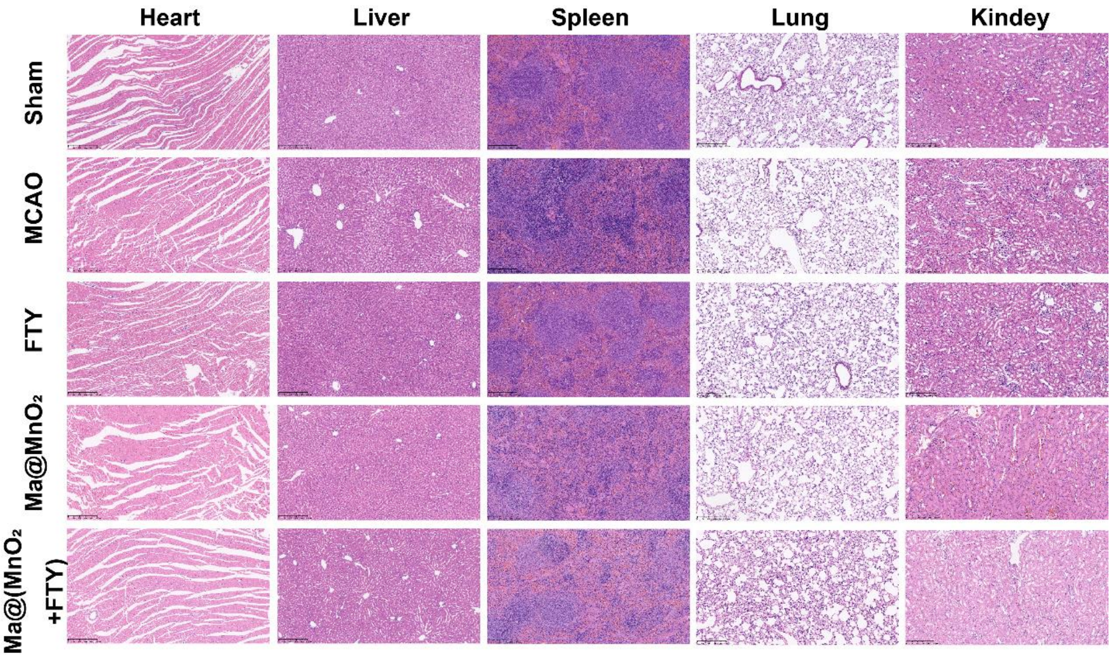

Figure S32. Representative images of main organs with H&E staining after the tMCAO/R rats were treated with different formulations (Scale bar, 250 μm).

Table S1. Pharmacokinetic parameters of free FTY and Ma@(MnO_2+FTY) nanoparticles at a dose of 1.5 mg Kg^{-1}. Data are presented as means ± SD, n = 3.

**Formulation**	**$AUC_{0-\infty}$ [mg/L×h]**	**MRT [h]**	**$t_{1/2}$ [h]**
Free FTY	2.285 ± 0.274	7.676 ± 0.437	14.597 ± 1.623
Ma@(MnO_2+FTY)	7.803 ± 1.841	16.680 ± 0.662	30.208 ± 2.804
